# TickSialoFam (TSFam): A Database That Helps to Classify Tick Salivary Proteins, a Review on Tick Salivary Protein Function and Evolution, With Considerations on the Tick Sialome Switching Phenomenon

**DOI:** 10.3389/fcimb.2020.00374

**Published:** 2020-07-24

**Authors:** José M. C. Ribeiro, Ben J. Mans

**Affiliations:** ^1^Section of Vector Biology, Laboratory of Malaria and Vector Research, National Institute of Allergy and Infectious Diseases, Rockville, MD, United States; ^2^Epidemiology, Parasites and Vectors, Agricultural Research Council - Onderstepoort Veterinary Research, Pretoria, South Africa; ^3^The Department of Veterinary Tropical Diseases, University of Pretoria, Pretoria, South Africa; ^4^Department of Life and Consumer Sciences, University of South Africa, Pretoria, South Africa

**Keywords:** tick, saliva, transcriptome, annotation, sialome switching, feeding, salivary glands

## Abstract

Tick saliva contains a complex mixture of peptides and non-peptides that counteract their hosts' hemostasis, immunity, and tissue-repair reactions. Recent transcriptomic studies have revealed over one thousand different transcripts coding for secreted polypeptides in a single tick species. Not only do these gene products belong to many expanded families, such as the lipocalins, metalloproteases, Antigen-5, cystatins, and apyrases, but also families that are found exclusively in ticks, such as the evasins, Isac, DAP36, and many others. Phylogenetic analysis of the deduced protein sequences indicate that the salivary genes exhibit an increased rate of evolution due to a lower evolutionary constraint and/or positive selection, allowing for a large diversity of tick salivary proteins. Thus, for each new tick species that has its salivary transcriptome sequenced and assembled, a formidable task of annotation of these transcripts awaits. Currently, as of November 2019, there are over 287 thousand coding sequences deposited at the National Center for Biotechnology Information (NCBI) that are derived from tick salivary gland mRNA. Here, from these 287 thousand sequences we identified 45,264 potential secretory proteins which possess a signal peptide and no transmembrane domains on the mature peptide. By using the psiblast tools, position-specific matrices were constructed and assembled into the TickSialoFam (TSF) database. The TSF is a rpsblastable database that can help with the annotation of tick sialotranscriptomes. The TSA database identified 136 tick salivary secreted protein families, as well as 80 families of endosomal-related products, mostly having a protein modification function. As the number of sequences increases, and new annotation details become available, new releases of the TSF database may become available.

## Introduction

Tick salivary glands serve multiple physiological roles: While off their hosts, special acini (type I) produce a hygroscopic saliva that spreads over the ticks' palps, absorbs atmospheric water vapor, and when re-ingested helps to keep the tick hydrated (Bowman and Sauer, 2004). The salivary glands are also a major excretion/water balance organ in ixodid ticks, as the majority of the water ingested during a blood meal is pumped back into their hosts as saliva (Binnington and Kemp, 1980). In ixodid males, due to their peculiar and successful oral sex reproduction mode, special male acini (type IV) contribute to salivary products that are uniquely male and are molecular homologs of insect seminal gland or vertebrate prostate gland proteins (Tan et al., 2015). Finally, and the focus of this article, tick salivary glands help blood feeding by secreting products that help tick attachment to the host's skin and that inhibit host hemostasis (a physiological response that encompasses platelet aggregation, vasoconstriction, and blood clotting), inhibit the vertebrate tissue repair response, and modulate host immunity (Francischetti et al., 2009).

There are 899 known tick species divided into two major families, the soft tick (*Argasidae*) and the hard tick (*Ixodidae*) families (Guglielmone et al., 2010). A third family, *Nuttalliellidae*, exists, but with a single species. Soft ticks feed relatively rapid (typically <1 h), while hard ticks feed for several days or weeks. Adult soft ticks can feed many times, while adult hard ticks feed only once. Hard ticks are further divided into Prostriate and Metastriate. Metastriate ticks have relatively short mouthparts, producing copious amounts of cement, while Prostriate ticks have longer mouthparts, producing less abundant cement. Adult and nymphal soft ticks do not produce cement. Blood is the only nutritious food for all ticks. It is estimated that there are over 15,000 species of arthropods that feed on blood, and this mode of feeding evolved independently at least 20 times (Mans, 2011)—thus creating a scenario of convergent evolution. Ticks, mosquitoes, sand flies, kissing bugs, and fleas evolved blood feeding independently, but they all share the presence of a salivary apyrase activity, an enzyme (ATP-diphosphohydrolase) that breaks down ATP and ADP released by damaged cells and trigger platelet and neutrophil activation. However, three different gene families were recruited for this task: (1) the 5′-nucleotidase in ticks (Stutzer et al., 2009), some genera of triatomine bugs, and mosquitoes; (2) the Cimex-type apyrase (CD73) in sand flies and the kissing bug genus *Rhodnius*; (3) the CD39 apyrase in fleas (Ribeiro and Arca, 2009). These types of enzymes are ubiquitous in eukaryotes, normally extracellular and bound to the membrane or intracellular and associated to endosomes; their transcripts always display a signal peptide indicative of secretion. Evolution of salivary apyrase, as an adaptation to blood feeding, thus happened by processes of gene duplication of a salivary gland expressed gene with loss of its membrane anchor (Champagne et al., 1995). Another common evolutionary process of adaptation to blood feeding involved further gene duplication events of already established salivary genes, which initially increased the mRNA dosage, but allowed for further functional or antigenic diversification. For example, sand flies and mosquitoes have several related salivary expressed genes of the D7 family, which belong to the odorant binding protein group, and are associated with binding of agonists of hemostasis and inflammation (Valenzuela et al., 2002a; Mans et al., 2007; Calvo et al., 2009; Alvarenga et al., 2010). Ticks and blood sucking Hemiptera had a large expansion of the lipocalin family, which are also associated with binding of agonists of hemostasis and inflammation (Andersen and Ribeiro, 2017). Fleas expanded members of the acid phosphatase family (function unknown) (Andersen et al., 2007; Ribeiro et al., 2012).

While Adult sand fly saliva has <50 polypeptides and mosquito saliva has near 100, hard tick saliva has several hundred, or thousands of polypeptides (Ribeiro and Arca, 2009; Ribeiro et al., 2010). The increased number of components in hard tick saliva was initially thought to derive from the prolonged period of tick feeding, where it would face not only the hemostatic host response, but also its tissue repair and immune responses. However, deep sequencing of salivary transcriptomes at different times of feeding uncovered that ticks change their salivary repertoire frequently, within hours; a process named “sialome switching” (Valenzuela et al., 2002b; Karim and Ribeiro, 2015; Perner et al., 2018). Thus, while in mosquitoes and other non-tick hematophagous organisms, all members of a salivary protein family are simultaneously expressed; in hard ticks, individual genes of the same family, or paralogs, are expressed at different times during the feeding process. Accordingly, when the host mounts an antibody response to a particular antigen, a process that takes a few days, chances are that the antigenic molecule has been substituted by a not antigenically recognizable paralogs. Sialome switching is thus thought to be a mode of immune evasion. It may also serve ticks when they feed on different host species, as they may “find” the best sialome when feeding on a lizard, which may be different then when feeding on a mouse. Sialome switching may thus be a mechanism adapting ticks to feed on different hosts.

The study of the function of saliva in blood feeding by arthropods in the last 30 years was transformed by the revolution of cheap DNA sequencing. In the beginning it was the “grind and find” way: salivary homogenates were used to discover activities determined by a bioassay; followed by chromatographic methods of purification of the biological activity; in the case it was a polypeptide, obtaining Edman sequences allowing the construction of degenerate DNA probes that were used to amplify fragments of the salivary cDNA; that would be used to find, by hybridization, and with luck, a full length clone from which the primary sequence of the studied protein could be determined. The research flow was then from the bioassay to the protein, and from the protein to the DNA or mRNA.

Currently, for <300 $, we can obtain 20 million sequences of 150 nucleotides (nt) in length that can be assembled “*de novo*” (in the absence of a genomic sequence to serve as an assembly scaffold), producing high quality transcript sequences that can be converted to their coding protein sequences. Accordingly, the “sialome” (from the Greek σíελoς = saliva) can be obtained inexpensively and in a relatively short amount of time (Ribeiro and Francischetti, 2003). These sialomes have revealed a surprising number of novel protein sequences; they frequently have no similar matches when comparing their primary sequence to a bank of known proteins. Less frequently, they produce matches to ubiquitous protein families, usually enzymes, but also members of the antigen-5 family or families of protease inhibitors, such as those containing cystatin, serpin, Kazal, or Kunitz domains. These deducted protein sequences can be further used in two ways: First, in the absence of the organism's genomic sequences, they will serve as a data bank for proteomic studies, a technology that requires a reliable “a priori” defined set of sequences; the tandem mass spectrometry (MS/MS) protocol maps the partial sequences.

Theoretically, if the genome sequence of the organism under study is known, then its deducted protein sequences could serve this purpose. However, most blood sucking insects and ticks still do not have a reliable genome sequence. The genome of the tick *Ixodes scapularis* is at best 50% complete (Gulia-Nuss et al., 2016). Even with better genomic data information, such as the anopheline mosquitoes (Holt et al., 2002; Neafsey et al., 2015), a detailed comparison of the protein sequences known to be salivary expressed with the deducted genomic protein sequences indicated that there was an error on ~50% of the predicted genomic sequences; either because they were missed, or because the intron-exon boundaries were incorrect (Arca et al., 2017). Proteomic studies of the salivary glands of blood sucking arthropods thus rely on carefully curated protein sequences deducted from the salivary transcriptome assembly. Second, the deducted protein sequences can guide production of recombinant proteins to be tested in bioassays to determine their functions, or as immunological markers of vector exposure. Thus, the research flows today from the mRNA to the protein, and from the protein to the bioassay.

Another approach exists, namely the immuno-proteome method, which is based on high throughput cloning and sequencing methods aimed at anti-tick vaccine discovery (Das et al., 2001; de la Fuente et al., 2006; Narasimhan et al., 2007; Radulovic et al., 2014; Becker et al., 2015; Lewis et al., 2015; Garcia et al., 2017). First, an animal is made hyperimmune to tick salivary proteins by previous tick exposure, or injection of salivary homogenates. The hyperimmune serum, and its control, are then used to scan expression cDNA libraries made from tick salivary glands. The positive clones are then sequenced, recombinantly expressed, and tested for their biological activity and as a vaccine to disrupt tick feeding. It has succeeded in discovering and characterizing many tick salivary proteins (Narasimhan et al., 2007; Dai et al., 2009; Schuijt et al., 2010). Somewhat surprisingly, many intracellular tick proteins, not having a signal peptide indicative of secretion, were discovered to be good antigen candidates; as was the case of the conserved tick protein, subolesin (de la Fuente et al., 2006), a member of the Akirin transcription regulator (de la Fuente et al., 2011). Similarly, a tick protein similar to vertebrate histamine releasing factor was found (Mulenga et al., 2003), and named histamine release factor (HRF). This is also a conserved member of the cytoskeletal family named “Translationally controlled tumor protein” (TCTP) and was responsible for the activity of human tissue homogenates to trigger histamine release by mast cells (Xiao et al., 2017). Human TCTP thus has a dual function: as a cytoskeletal protein: and as a cytokine-like protein when cells lyze and their contents are released to the extracellular milieu. ADP and ATP similarly are primarily intracellular molecules; however when released to the extracellular compartment after cellular disruption or secretion, they similarly trigger pro inflammatory reactions. It thus appears that tick saliva contains proteins that are secreted through the classic merocrine pathway, as well as non-conventional secretion pathways, including apocrine or holocrine secretions (Farkas, 2015). Indeed, merocrine and apocrine secretion has been characterized by ultrastructural studies in soft tick salivary glands (Coons and Roshdy, 1981). Hard tick salivary glands suffer a degeneration process after a blood meal (Harris and Kaufman, 1981; Friesen and Kaufman, 2009), and it is possible that this process starts while the tick is still feeding and thus generating a holocrine salivary secretion. Additionally, there is recent evidence that exosomes may be secreted in tick saliva (Hackenberg and Kotsyfakis, 2018; Zhou et al., 2018; Chavez et al., 2019). Exosomes can fuse with host cells and deliver their products intracellularly, including microRNAs that may inhibit translation of some protein types (Keller et al., 2006). The study of salivary exosomal secretion in tick feeding is still in its infancy, and the next decade should uncover its more precise role in feeding by ticks.

**Figure 1:**
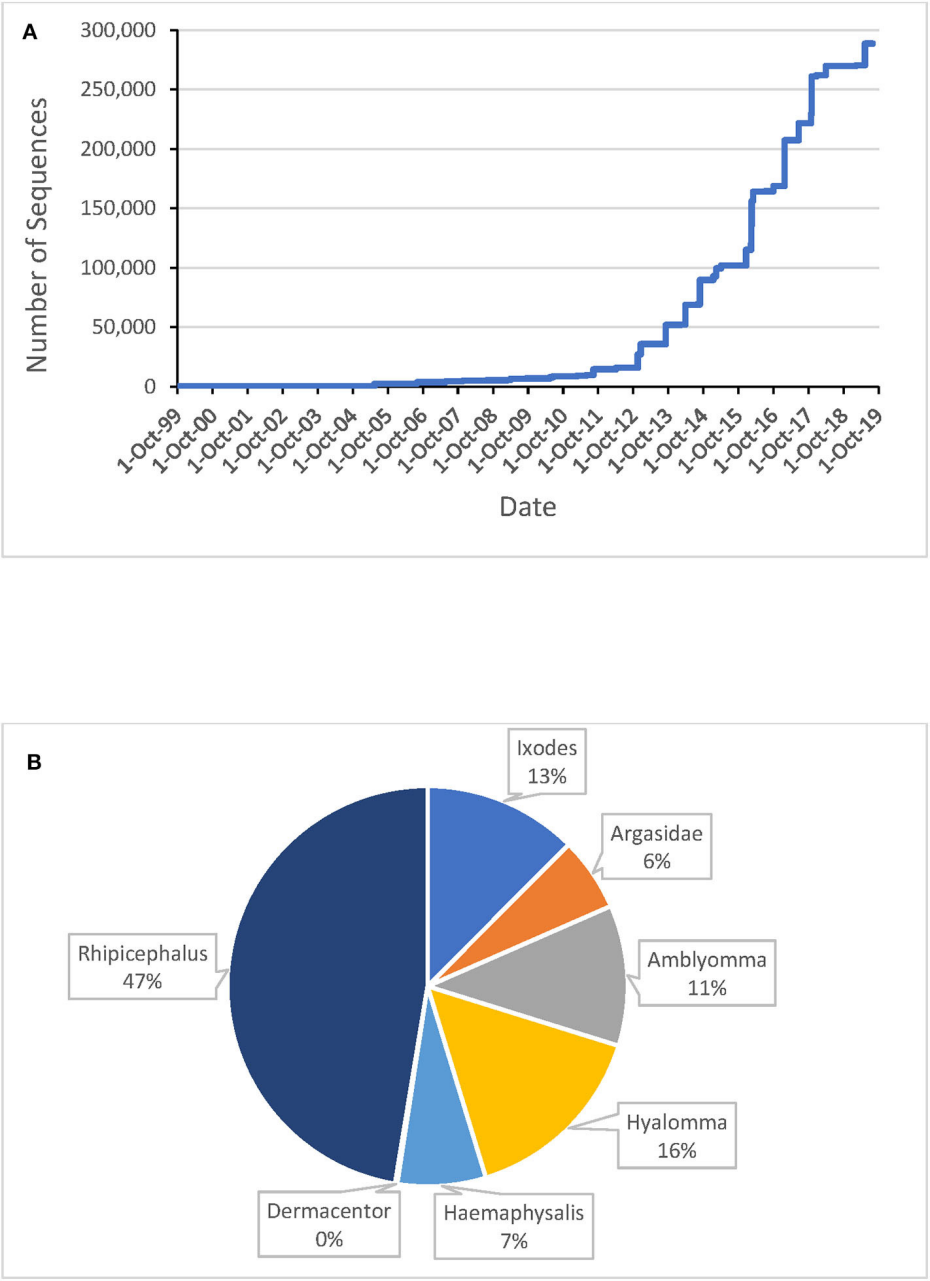
Tick salivary protein coding sequences (excluding EST's) deposited at the National Center for Biotechnology Information (NCBI). **(A)** Number of sequences deposited, 1993–2019. **(B)** Breakdown of sequences available in Nov/2019 by genus or family.

Ten years ago, a review on tick sialomes based on 3,500 tick salivary proteins identified more than 30 protein families, most having unknown function (Francischetti et al., 2009). That review was done when the first sialomes were uncovered with the now obsolete Sanger DNA sequencing method. At that point in time, the now defunct 454 and surviving Illumina protocols appeared, and the number of salivary-derived tick protein-coding sequences deposited to the National Center for Biotechnology Information (NCBI) soared, reaching over 287,000 in November/2019 (Figure 1). These sequences derive from 44 species within 10 genera; only 19 species from 7 genera are represented with more than one thousand sequences (Table 1). Considering that there are 899 tick species from 20 different genera (Guglielmone et al., 2010), we have achieved 1,000 sequences for only 2.7% of the known tick species, which covers 35% of the known genera. Clearly, we are still far from knowing the complete sialome repertoire of ticks (the sialoverse), a task that should take one or more decades for its fulfillment.

**Table 1:** Tick salivary coding sequences retrieved from NCBI on November 2019.

**A**		**B**	
**Species**	**Number of sequences**	**Species**	**Number of sequences**
*Amblyomma americanum*	3,967	*Rhipicephalus appendiculatus*	49,146
*Amblyomma aureolatum*	93	*Rhipicephalus bursa*	39,435
*Amblyomma cajennense*	7,098	*Hyalomma dromedarii*	38,518
*Amblyomma maculatum*	4,855	*Ixodes ricinus*	33,250
*Amblyomma parvum*	2,838	*Rhipicephalus annulatus*	20,753
*Amblyomma rotundatum*	355	*Haemaphysalis longicornis*	20,592
*Amblyomma sculptum*	4,249	*Rhipicephalus zambeziensis*	14,393
*Amblyomma triste*	8,098	*Rhipicephalus pulchellus*	11,227
*Amblyomma tuberculatum*	67	*Amblyomma triste*	8,098
*Amblyomma variegatum*	743	*Ornithodoros turicata*	7,560
*Antricola delacruzi*	317	*Amblyomma cajennense*	7,098
*Argas monolakensis*	1,059	*Ornithodoros rostratus*	6,587
*Carios mimon*	4	*Hyalomma excavatum*	5,338
*Dermacentor andersoni*	505	*Amblyomma maculatum*	4,855
*Haemaphysalis longicornis*	20,592	*Amblyomma sculptum*	4,249
*Haemaphysalis qinghaiensis*	2	*Amblyomma americanum*	3,967
*Hyalomma anatolicum anatolicum*	88	*Amblyomma parvum*	2,838
*Hyalomma asiaticum asiaticum*	1	*Ixodes scapularis*	2,451
*Hyalomma dromedarii*	38,518	*Argas monolakensis*	1,059
*Hyalomma excavatum*	5,338	*Rhipicephalus microplus*	969
*Hyalomma rufipes*	558	*Rhipicephalus sanguineus*	761
*Ixodes affinis*	1	*Amblyomma variegatum*	743
*Ixodes holocyclus*	7	*Hyalomma rufipes*	558
*Ixodes pacificus*	118	*Ornithodoros parkeri*	544
*Ixodes persulcatus*	17	*Dermacentor andersoni*	505
*Ixodes ricinus*	33,250	*Ornithodoros brasiliensis*	440
*Ixodes scapularis*	2,451	*Amblyomma rotundatum*	355
*Ixodes sinensis*	5	*Antricola delacruzi*	317
*Ornithodoros brasiliensis*	440	*Ornithodoros coriaceus*	272
*Ornithodoros coriaceus*	272	*Ixodes pacificus*	118
*Ornithodoros kalahariensis*	2	*Amblyomma aureolatum*	93
*Ornithodoros moubata*	59	*Hyalomma anatolicum anatolicum*	88
*Ornithodoros parkeri*	544	*Amblyomma tuberculatum*	67
*Ornithodoros rostratus*	6,587	*Ornithodoros moubata*	59
*Ornithodoros turicata*	7,560	*Ixodes persulcatus*	17
*Rhipicephalus annulatus*	20,753	*Ixodes holocyclus*	7
*Rhipicephalus appendiculatus*	49,146	*Ixodes sinensis*	5
*Rhipicephalus bursa*	39,435	*Carios mimon*	4
*Rhipicephalus haemaphysaloides*	1	*Haemaphysalis qinghaiensis*	2
*Rhipicephalus microplus*	969	*Ornithodoros kalahariensis*	2
*Rhipicephalus pulchellus*	11,227	*Hyalomma asiaticum asiaticum*	1
*Rhipicephalus sanguineus*	761	*Ixodes affinis*	1
*Rhipicephalus zambeziensis*	14,393	*Rhipicephalus haemaphysaloides*	1
Total	287,343	Total	287,343

***(A)** Data sorted by species and **(B)** data sorted by number of sequences*.

To facilitate the task of annotating newly assembled transcriptomes, we present a rpsblastable database of tick salivary families, named TickSialoFam or TSFam. Together with an update on the classification of tick salivary families as proposed in 2009 (Francischetti et al., 2009), we provide further insights into the evolution of the genes coding for these proteins.

## Methods

### Sequence Retrieval and Organization

The nucleotide database from NCBI (https://www.ncbi.nlm.nih.gov/nuccore/) was queried with the expression “ixodida[organism] AND salivary,” and the resulting records were downloaded in the gb (GenBank) format. Sequences with “mitochondr” in their description were excluded, as well as those results related to unassembled EST's, and those from the REFSEQ database. This resulted in a set of 287,343 sequences (Table 1). It should be noted that up to mid-2018, all protein sequences submitted to the NCBI Transcriptome Shotgun Annotation (TSA) database were deposited in the NCBI protein database, as were the nucleotide sequences deposited to the NCBI nucleotide database. After this time, all TSA submissions appear only in the nuccore database, solely as a link leading to downloading the nucleotide and protein sequences. These TSA databases were then downloaded and added to those of the nuccore database. A script written in visual basic version 6 (VB6) extracted the individual protein sequence in fasta format; this script also built a table that included the sequence accession code, its description, author's, date of publication of the sequence, and bibliographical information (including link to PubMed, when available). When the protein sequence was unavailable, the larger open reading frame was translated. These data were imported into a hyperlinked Excel spreadsheet available as Supplemental Spreadsheet 1.

Since this sequence set contained both those related to housekeeping, as well as a salivary secreted function (from now on just called as “housekeeping” or “secreted,”) we ran these sequences through the SignalP program (version 3.0) (Nielsen et al., 1999) to identify whether they had a predicted signal sequence indicative of secretion. We also ran them as well as through the TMHMM program (Sonnhammer et al., 1998) to determine their predicted transmembrane domains. Of the 287,343 sequences, 45,264 had a signal sequence, excluding those having one or more transmembrane domain outside the signal sequence; thus obtaining a set coding for putative classical secreted proteins (Table 2). To identify protein families, we clusterized these sequences by blasting each one against the remaining set and joining those that reached variable degrees of identity (varying from 25 to 95% in 5% increments), over at least 75% of the length of the larger sequence. Notice that if a polypeptide A satisfies the rule with polypeptide B, and B with polypeptide C, the cluster ABC would be formed, even though polypeptide A does not satisfy the rule with polypeptide C. For each cluster, we obtained a fasta file with its members. In the cases where the cluster had 5 or more sequences, a clustal (Larkin et al., 2007) alignment was obtained. The resulting fasta files and clustal alignments were hyperlinked to the spreadsheet with the indication of the cluster number (ordered from the most to the least abundant in sequences) and its number of sequences (Supplemental Spreadsheet 2).

**Table 2:** Putative secreted class of tick salivary sequences retrieved from NCBI on November 2019.

**A**		**B**	
**Species**	**Number of sequences**	**Species**	**Number of sequences**
*Amblyomma americanum*	1,369	*Ixodes ricinus*	9,776
*Amblyomma aureolatum*	27	*Rhipicephalus appendiculatus*	6,690
*Amblyomma cajennense*	2,072	*Hyalomma dromedarii*	3,833
*Amblyomma maculatum*	917	*Rhipicephalus bursa*	3,190
**Amblyomma parvum**	721	*Rhipicephalus zambeziensis*	3,017
*Amblyomma rotundatum*	164	*Amblyomma triste*	2,542
*Amblyomma sculptum*	307	*Haemaphysalis longicornis*	2,493
*Amblyomma triste*	2,542	*Amblyomma cajennense*	2,072
*Amblyomma tuberculatum*	26	*Rhipicephalus pulchellus*	1,969
*Amblyomma variegatum*	267	*Rhipicephalus annulatus*	1,467
*Antricola delacruzi*	83	*Amblyomma americanum*	1,369
*Argas monolakensis*	301	*Amblyomma maculatum*	917
*Dermacentor andersoni*	154	*Ixodes scapularis*	890
*Haemaphysalis longicornis*	2,493	*Ornithodoros turicata*	818
*Hyalomma anatolicum anatolicum*	27	*Amblyomma parvum*	721
*Hyalomma asiaticum asiaticum*	1	*Ornithodoros rostratus*	657
*Hyalomma dromedarii*	3,833	*Hyalomma excavatum*	397
*Hyalomma excavatum*	397	*Amblyomma sculptum*	307
*Hyalomma rufipes*	133	*Argas monolakensis*	301
*Ixodes affinis*	1	*Amblyomma variegatum*	267
*Ixodes holocyclus*	7	*Rhipicephalus microplus*	257
*Ixodes pacificus*	74	*Rhipicephalus sanguineus*	224
*Ixodes persulcatus*	14	*Amblyomma rotundatum*	164
*Ixodes ricinus*	9,776	*Ornithodoros parkeri*	162
*Ixodes scapularis*	890	*Dermacentor andersoni*	154
*Ixodes sinensis*	5	*Hyalomma rufipes*	133
*Ornithodoros brasiliensis*	61	*Ornithodoros coriaceus*	111
*Ornithodoros coriaceus*	111	*Antricola delacruzi*	83
*Ornithodoros kalahariensis*	2	*Ixodes pacificus*	74
*Ornithodoros moubata*	37	*Ornithodoros brasiliensis*	61
*Ornithodoros parkeri*	162	*Ornithodoros moubata*	37
*Ornithodoros rostratus*	657	*Amblyomma aureolatum*	27
*Ornithodoros turicata*	818	*Hyalomma anatolicum anatolicum*	27
*Rhipicephalus annulatus*	1,467	*Amblyomma tuberculatum*	26
*Rhipicephalus appendiculatus*	6,690	*Ixodes persulcatus*	14
*Rhipicephalus bursa*	3,190	*Ixodes holocyclus*	7
*Rhipicephalus haemaphysaloides*	1	*Ixodes sinensis*	5
*Rhipicephalus microplus*	257	*Ornithodoros kalahariensis*	2
*Rhipicephalus pulchellus*	1,969	*Hyalomma asiaticum asiaticum*	1
*Rhipicephalus sanguineus*	224	*Ixodes affinis*	1
*Rhipicephalus zambeziensis*	3,017	*Rhipicephalus haemaphysaloides*	1
Total	45,264	Total	45,264

***(A)** Data sorted by species and **(B)** data sorted by number of sequences*.

From the original set of 287,343 sequences, we extracted the 45,264 sequences classified as secreted. The remaining sequences were submitted to the secretomeP program to identify those sequences that could qualify as secreted through non-classical pathways, as indicated by a secretomeP score larger than 0.6 (Bendtsen et al., 2004). The resulting 125,197 sequences are displayed on Supplemental Spreadsheet 3.

### TickSialoFam Model Construction

A program was written in VB6 that inspected each alignment file from the various clusters of the “Secreted” data set (Supplemental Spreadsheet 2). In the cases where it had five or more sequences, and if the alignment indicated at least five sites of identity or conservation, then the fasta file was used to construct a PSIBlast-based model (Altschul et al., 1997; Schaffer et al., 2001). First, all sequences of the cluster were blasted against themselves, and the one that accrued the largest sum of scores (excluding self-blast) was elected as the cluster centroid. The program blastpgp, from the blast package (Psi-blast), was then run using the centroid sequence as input; the formatted cluster fasta sequences (using the blast suit program formatdb) was used as a target databank, with the parameters -W 2 (blastp word size), and -j 5 (maximum number of iterations). The -h switch (inclusion *e* value after the first blast step) was variable, according to the size of the centroid: the chosen values were 1e-15 for length (l) larger than 200; 1e-8 for 100 < l <200; 1e-6 for 60 < l <100; and 1e-4 for l <60. These values were arbitrarily adjusted according to size of the sequence: because a smaller sequence accrues a smaller score value than a large sequence and thus has a higher e value. The resulting matrix file was saved using the -C switch. These models were named according to the cluster parameters; for e.g., a model named 35-123 derived from the sequences found in cluster number 123 from the clusterization at 35% identity. These procedures are similar to those used for construction of the COG and KOG databases (Tatusov et al., 2003).

Another method to create matrices was done by psiblasting each sequence against the whole Ixodida database, using the same parameters indicated above. These models are named by the NCBI accession number of the parent protein sequence. These matrix files were then combined with the program makeprofiledb with the parameters -threshold 9.82 -scale 100.0 -dbtype rps -index true, forming the initial TSFam database. Finally, the number of models used in the final database was reduced by selecting those that produced a match with a maximum *e*-value of 1e-20.

### Sequence Functional Classification

To further help classification of the protein sequences, possible proline-hydroxylation (Rhoads and Udenfriend, 1969; Bohmer, 1971; Kivirikko et al., 1972) and tyrosine sulfation (Nicholas et al., 1999) sites were determined, including the percentages of glycines, prolines, and tyrosines of each protein sequence, and their number of cysteine residues. These features helped to classify the families within the glycine-rich proteins (GRP) group. Determination of the presence of glycosyl-phosphate-inositol (GPI) anchors were determined with the program DGPI (Pierleoni et al., 2008). This helped to identify secreted proteins that were bound to membranes. The presence of furin cleavage sites were also detected (Duckert et al., 2004), identifying polyproteins with various cleavage sites. The percentages of serines and threonines were determined. The number of possible mucin-type galactosylation sites being measured with the program NetOGlyc (Hansen et al., 1998; Julenius et al., 2005) helps to further identify and classify the mucins. The set of secreted sequences were compared by blastp; to a subset of proteins from the NCBI NR database; to the Swissprot database; to the Enzyme Commission set from the KEGG database; to the salivary Ixodida sequences retrieved as indicated above; to the top 100 proteins associated with exosomes from exocarta (http://exocarta.org/download); and to rpsblast comparisons to the Conserved Domains database (CDD), Kog, Pfam, Smart, and TSF databases. All these comparisons were hyperlinked to the corresponding spreadsheets and allowed manual annotation of the sequences.

The annotations were transferred to the TSF models, which included in their description the following fields of information: (1) model name: as indicated in the previous section (e.g., 35-123 or JAP81818.1). (2) Group: This classification may include non-phylogenetically related sequences, such as the glycine-rich proteins (GRP) and mucins but can include groups such as the Lipocalins or Serpins. For this reason, this field is named group and not family. (3) Family: A subdivision of the group, containing phylogenetically related sequences. This field is empty in many models. (4) H/S/I: Indicates whether the sequence is classified as Secreted, Housekeeping, or in a few cases, Indeterminate. (5) Other: Includes additional information codified as E, enzyme; AM, antimicrobial; and PI, protease inhibitor. These fields are separated by “|” characters, which may be changed to tabs to produce the display shown in the hyperlinked spreadsheets.

The publicly available formatted TSFam database (Supplemental File 1) can be used in a rpsblast search using as an input a fasta file of tick salivary proteins, using a command line such as: “[x]blastall -p rpsblast -d [y]TSF -i [z]in.fasta -o [z]tsf.blt -IT -JT -v20 -b10 -e1e-4 -FF.” Where [x], [y], and [z] are the paths to the blast program, the TSFam database, and the input fasta and the output files, respectively. We recommend accepting the classifications using the TSFam database when the coverage of the model by the queried protein is at least 2/3 (>66.6%) and having a maximum *e* value of 1e-4. To access the blast programs, use version 2.2.26 available at ftp://ftp.ncbi.nlm.nih.gov/blast/executables/legacy.NOTSUPPORTED/2.2.26/.

### Tools That Help With Mining the Database

Three tools, written in visual basic and compiled to run under the Windows environment, are available upon email request to jribeiro@niaid.nih.gov. The tool, fasta2tbl, reads a fasta file and creates a hyperlinked table that can be imported into a spreadsheet. The tool, rpsblast2tbl, reads a rpsblast result file and maps the results and annotation into a hyperlinked table that can be imported into the fasta table done with fasta2tbl. The tool, GetFSA, takes a list of sequence names found in the fasta table or spreadsheet and creates a fasta file that can be used for phylogenetic or other studies.

### Phylogenetic Analysis

Phylogenetic analysis were done with Mega7 (Kumar et al., 2016). Alignments were done with Clustal (Larkin et al., 2007) and refined with Muscle (Edgar, 2004) using the MEGA7 program (Kumar et al., 2016). The program FUBAR (Murrell et al., 2013) of the HYPHY (Pond et al., 2005) package was used to find positively selected sites within the in-frame nucleotide sequences. Positively selected codons were identified by their assigned probabilities of the parameter α being smaller than beta. The program RDP4 was used to determine the number of recombination breakpoints within the nucleotide alignments (Martin and Rybicki, 2000).

## Results

Table 1 lists the number of retrieved sequences by species, sorted by species name (A), and number of sequences (B); Table 2 lists the same information for the subset of sequences classified as “secreted.”

Because the sequences under this study derive mainly from transcriptome assembly studies, many of the sequences are truncated either at their 5′ or 3′ ends. Only 57% of the 45,264 protein sequences start with a methionine (and even if it starts with one, there is no guarantee that it is the starting methionine); stop codons are found for 57% of the sequences. Both a starting methionine and stop codon occur in 49% of the sequences. Accordingly, identification of signal peptide indicative of secretion can only be found with confidence in a maximum of 57% of the set. Despite these flaws, truncated protein sequences are still important, as they may later serve as sequence templates for MS/MS studies that may include extension of selected coding sequences.

### Putative Secreted Proteins

**Figure 2:**
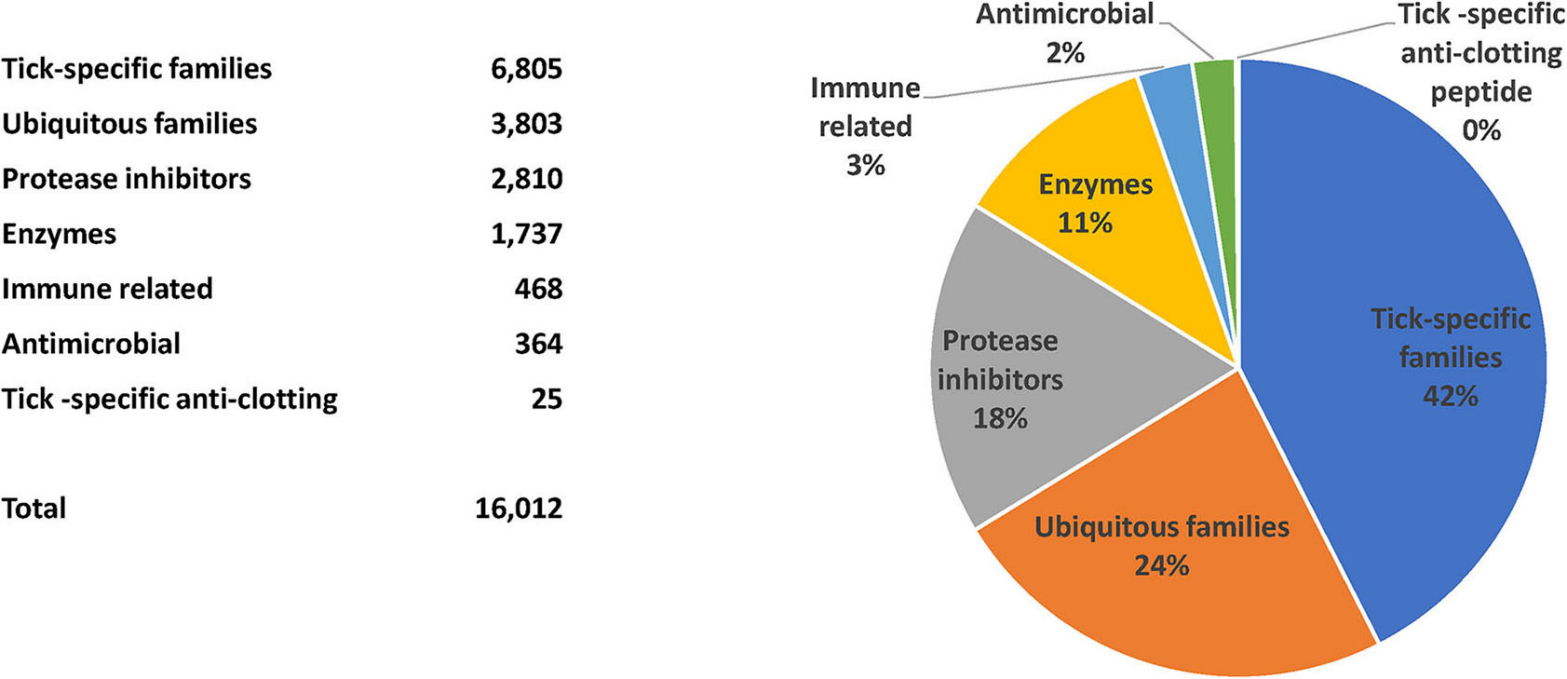
Classification and relative number of sequences for the putative secreted salivary proteins from ticks.

**Figure 3:**
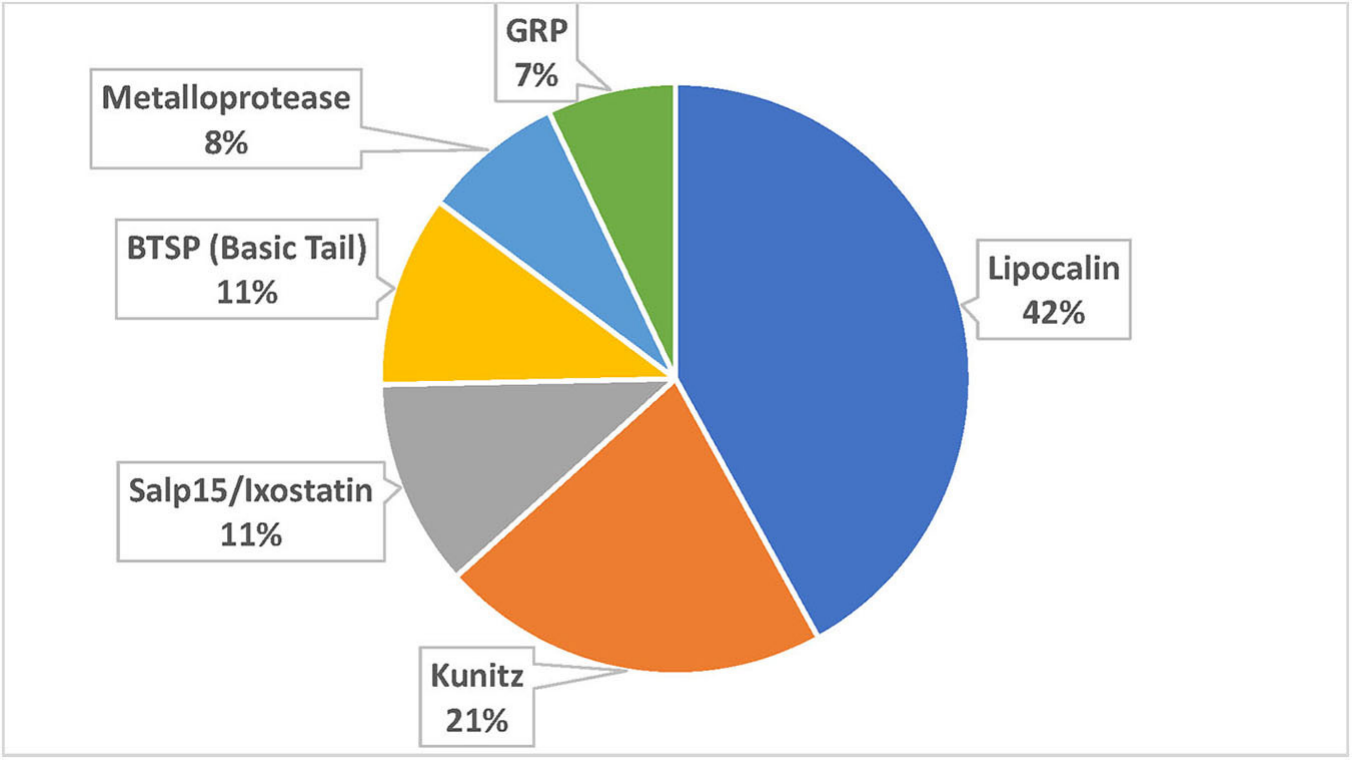
Proportion of sequences accrued by the six larger superfamilies.

The TSFam database of motifs allowed classification of 18,963 sequences from the 45,264 salivary-derived Ixodida sequences from NCBI, classified as putative secreted proteins (Supplemental Spreadsheet 2). These were organized into 136 groups, as indicated in the Supplemental Table 1. The criteria for classification of these sequences required an *e*-value <1e-4 and a model coverage of at least 67% (2/3 of the model length). The classifications can be further grouped according to their similarities, or lack of similarities, to other known proteins or protein domains; they can also be grouped according to their known function, leading to the categories “Enzymes,” “Protease inhibitors,” “Ubiquitous families,” “Tick-specific anti-clotting peptides,” “Immune-related,” “Antimicrobials,” and “Tick-specific families” (Figure 2). Notice that the category “Tick-specific families” is the larger one, however, most of its members have no known function. Many of the groups are complex in nature and may contain phylogenetically unrelated families; for instance, the “Glycine-rich family,” is further subdivided into 19 families in Supplemental Spreadsheet 2. Six of the groups contains the majority of sequences (Figure 3).

Considering all 136 groups of Supplemental Table 1, only 37 have been characterized with at least one member having been recombinantly expressed and its function determined by bioassay, enzymatic assay, or proteomic detection from tick saliva. These studies are referenced in Supplemental Table 1. From these 37 characterized families, four have been only enzymatically characterized. Accordingly, the responsible tick protein is only presumed; nine have been found by saliva proteomic studies, but their function has not been characterized. It is also possible that many of the deducted CDS derive from pseudogenes (Kalyana-Sundaram et al., 2012). Thus, we have relative confidence that we know the molecular nature and function of at least one member from 24 of the 136 groups. Confidence should be defined in this case as: (1) Ascertaining that the protein is expressed in the saliva; (2) determining the biological activity of the recombinant protein. Very few protein families have at least one member satisfying these two requirements. Accordingly, this database consists mostly of a hypothetical set to be further characterized by proteomic and functional studies and should be updated as new studies are published.

This new data set provides the mining rounds for further analysis into the evolution of previously known families, including characterization of novel protein families by recombinant expression and functional assay of the predicted proteins.

### Evolutionary Insights on Tick Salivary Proteins

What follows is the phylogenetical analysis of selected protein families. Some large families have complex composition (such as, Kunitz, basic tail, Salp15) and will not be analyzed.

### Apyrase/5′-Nucleotidase Family

As stated in the introduction, the saliva of unrelated blood feeding arthropods has the ability to hydrolyze ADP and ATP, thus inhibiting important pathways of platelet and neutrophil aggregation and other pro-inflammatory reactions (Francischetti et al., 2009). The Supplemental Spreadsheet 2 and Table 1 provide for 50 full length sequences belonging to the 5′-nucleotidase family; four of which have a GPI anchor, indicating they are not secreted and could represent the homolog ancestral genes that gave origin to gene versions coding for secreted products. Supplemental Spreadsheet 1 indicate the presence of 287 annotations for 5′-nucleotidase; fifty of which are full-length. Phylogenetic analysis of these 50 sequences (Supplemental Figure 1) confirms this scenario: The GPI-containing sequences group together in a single clade, while the remaining sequences group into three major clades, Clades I and II, with Metastriate and Prostriate sequences, and an Argasidae clade. Clade I has two main branches, one containing Prostriate sequences and another containing only Metastriate sequences. The Prostriate sub clade further divides into two branches, indicative of an ancient gene duplication event. Clade II is similarly organized, with two main branches, and containing a single Prostriate sequence. It is also relevant that the depth of amino acid divergence on the clades I and II are at least 2-fold of the depth of the classic GPI clade, which contains one sequence from *Ixodes, two* from *Rhipicephalus*, and one from *Amblyomma*. Notice that the analysis has many more sequences derived from *I. ricinus* than from other species, reflecting the larger read coverage for the *Ixodes* transcriptomes. As coverage is increased for other species, novel genes coding for products of the 5′-nucleotidase/Apyrase should appear. The phylogram indicates the existence of at least seven genes coding for secreted apyrases from *I. ricinus*, based on the branches having 100% bootstrap support; the amino acid divergence of the secreted proteins is larger than that of the housekeeping, GPI anchored sequences, indicating faster evolution of the genes coding for secreted salivary genes; this was either caused by neutral evolution of these genes when compared to a purifying evolution scenario of the housekeeping genes, or due to positive selection pressure on the genes coding for secreted enzymes, driven by the immune pressure of their hosts (Mans et al., 2017). Submission of the apyrase coding enzymes to the FUBAR analysis (Murrell et al., 2013) indicated no sites under positive selection. RDP analysis (Martin and Rybicki, 2000) using the same sequence alignment indicated three recombination breakpoints (Supplemental Spreadsheet 4).

### Lipocalins

There are 3,689 sequences classified as lipocalins in Supplemental Spreadsheet 2 (considering >66% coverage with a TSFam lipocalin match). A subset of these sequences that had the PFAM His bind domain (with at least 80% coverage) amounted to 1,050 sequences. Attempts to construct a phylogeny (based on the faster Neighbor-Joining algorithm) with this subset were not informative, with most clades having poor bootstrap support. The phylogeny reconstruction of a smaller subset of 32 *I. ricinus* sequences, that were clustered at 40% sequence identity (Supplemental Figure 2), shows two main clades that further subdivide in eight smaller clades containing 2 or more sequences. FUBAR analysis indicated 16 codons under positive selection. RDP analysis indicated 11 recombination breakpoints (Supplemental Spreadsheet 4).

### Metalloproteases

The TSF algorithm indicates 677 sequences as being metalloproteases in Supplemental Spreadsheet 2. Eighty-seven of these sequences, from *I. ricinus, A. americanum*, and *R. appendiculatus* were chosen for alignment and phylogenetic analysis based on their completeness and match to the CDD domain cd04272 named ZnMc_salivary_gland_MPs. The cladogram displays five Metastriate-specific clades with strong bootstrap support, plus five Prostriate ones; each clade further subdividing in two or more subclades (Supplemental Figure 3). We can conclude that in *I. ricinus*, and possibly in other ticks, there are at least 10 genes coding for this subdivision of the salivary metalloproteases. FUBAR analysis found no sites under positive selection. RDP indicated 3 recombination breakpoints (Supplemental Spreadsheet 4).

### DAP36 Family

The founding member of this family was identified as an immunosuppressant salivary protein from *Dermacentor andersoni* (Bergman et al., 1995, 1998). In 2009 this family was characterized as a diverse Metastriate-specific family. However, here we find it is also composed of *Ixodes ricinus* members, as depicted in the phylogram of Supplemental Figure 4, built with a total of 121 sequences showing eight clades with strong bootstrap support, each containing subclades. These clades are composed of sequences originating from the same genus. FUBAR indicated strong positive selection for seven sites RDP analysis indicated six recombination breakpoints (Supplemental Spreadsheet 4).

### Cystatins

Cystatins are peptide inhibitors of cysteine proteases that are widespread in vertebrates (Abrahamson et al., 2003). They were first found in tick saliva by screening a salivary transcriptome of *I. scapularis* (Ribeiro et al., 2006). Different from lipocalins, metalloproteases, and Kunitz products, salivary cystatins are poorly transcribed. A recombinant peptide indicated its specificity for cathepsin L, and the protein was named sialostatin L. The expected activity was found in the tick saliva and expression of the protein was confirmed by reactive antibodies raised against the recombinant protein (Kotsyfakis et al., 2006). Naturally infested guinea pigs do not develop antibodies against sialostatin L, but vaccination of guinea pigs with sialostatin L reduced the feeding of ticks, and a boost reaction was observed (Kotsyfakis et al., 2008). A second *I. scapularis* salivary cystatin, named sialostatin L2, has been characterized (Chen et al., 2014; Lieskovska et al., 2015a). The effects of sialostatin L and L2 in mammalian immunity has been well-studied (Sa-Nunes et al., 2009; Kotsyfakis et al., 2010; Horka et al., 2012; Schwarz et al., 2012; Bruhl et al., 2014; Chen et al., 2014; Klein et al., 2015; Lieskovska et al., 2015a,b; Wang et al., 2016; Kotal et al., 2019). Supplemental Figure 5 depicts the phylogram of 82 tick cystatins, which indicates at least 17 clear clades each being mostly genus specific. FUBAR analysis does not recognize any codon site under positive selection, but RDP indicates the existence of one recombination breakpoint (Supplemental Spreadsheet 4).

### Proteins Possibly Secreted by Alternative Pathways

Supplemental Spreadsheet 3 contains proteins without a classical secretion signal but containing a secretomeP score indicative of secretion via non-classical pathways. Among these there are the subolesins, described in the introduction. Phylogenetic analysis, based on the alignments of 16 coding sequences, shows three clades of FUBAR indicates no sites under positive selection. RDP analysis indicates one recombination breakpoint.

### Housekeeping Proteins

Annotation of the SignalP-containing sequences in Supplemental Spreadsheet 2 by the TSA algorithm identifies 1,931 coding sequences, organized into 80 groups (Supplemental Table 2 and Supplemental Spreadsheet 2) that probably have a housekeeping function associated with the ER or Golgi compartments. Notably, 1,409 sequences representing 72% of the total group of housekeeping sequences are enzymes. Among these enzymes, 187 code for glycosyltransferases, most probably associated with glycosylating the salivary secretome. The glycosylation of tick salivary proteins has been under scrutiny recently, due to the epidemics of α-Gal allergy triggered by consumption of beef proteins (Commins and Platts-Mills, 2013). Human cases are associated with previous exposure to tick bites, and spatially correlated with tick abundance (Commins et al., 2011; Mateo-Borrega et al., 2019). A recent search for enzymes coding for α-Gal transferases (GALT) in ticks (Cabezas-Cruz et al., 2018) showed that the classical α1-3 GALTs that produce the Galα1-3Galβ1-(3)4GlcNAc-R (α-Gal) are absent in ticks; their function is taken by other transferases of the α1-4 and β1-4 GALT families. These enzymes are recognizable in Supplemental Spreadsheet 2 by the TSFam group of Glycosyltransferases and subfamily Lactosylceramide 4-alpha-galactosyltransferase. Other previously identified enzymes that are associated with protein modification (Ribeiro et al., 2006; Francischetti et al., 2009) include those promoting proline hydroxylation and tyrosine sulfation. While tyrosine sulfation has been determined in tick salivary anticlotting peptides (Thompson et al., 2017; Watson et al., 2019), proline hydroxylation, a common modification in collagen (Bohmer, 1971), has not been confirmed in ticks despite the high abundance of salivary collagen-like cement proteins that display the motifs triggering proline hydroxylation (Rhoads and Udenfriend, 1969; de Jong et al., 1991; Shimizu et al., 2005). Proteomic studies should include these possible modifications in the databases used for identification of peptide fragments obtained by MS/MS.

## Discussion

The TSFam database, and its associated hyperlinked spreadsheets of tick salivary proteins and associated nucleotide sequences, helps the annotation of tick salivary secreted proteins, as well as the retrieval of these sequences for phylogenetic studies. It has been reported that the genes coding for salivary proteins of blood sucking arthropods show high evolutionary rates; either due to the relaxed constraint of the genes allowing for less negative selection pressure, or for the more rare occurrence of positive selection (Daix et al., 2007; Schroeder et al., 2007; Decrem et al., 2008; Dai et al., 2012; Arca et al., 2014; Mans et al., 2017). The phylogenetic analysis, derived from the alignment of tick salivary coding sequences from the lipocalin, metalloprotease, cystatin, apyrase, DAP36, and subolesin families (Supplemental Figures 1–6 and Table 3), indicate a high number of recombination breakpoints on the genes coding for lipocalins (11 sites) and DAP36 (6 sites); three recombination breakpoint sites for the apyrase and metalloproteases; and one recombination breakpoint on the genes coding for the cystatins and subolesin. No positive selection sites were identified for the metalloprotease, cystatin, and subolesin genes. It is to be noted that the occurrence of recombination breakpoints could lead to false predictions of positive selected sites (Posada and Crandall, 2002; Bay and Bielawski, 2011). While positive selection is associated with fast gene evolution and divergence, intragenic recombination can strongly contribute to genome diversity among individuals, a situation that might be occurring within the lipocalin and DAP36 coding genes (Lauer et al., 2018; Olabode et al., 2019; Salim et al., 2019). As a note of caution, it should be considered that the diversity of transcripts from these multi-gene families could be artifactual due to the “*de novo*” assembly from small reads which could create false recombinant assemblies (Salmon et al., 2010). I A possible way to verify the frequency of these artifacts would be to compare the resulting lipocalin transcripts derived from single tick transcriptomes to those derived from the combined libraries. On the other hand, if recombination exists and leads to new protein variants, is it a classical meiotic recombination derived from a high number of intra-genic breakpoints, or does it occur in a somatic environment, such as occurs with vertebrate immunoglobulins This question could be answered by attempting the genome mapping of each transcript and identifying whether the transcript derives from a mosaic of exons or a “well-behaved” sequence of exons.

**Table 3:** Recombination breakpoints and positive selected sites predicted by RDP4 (Martin et al., 2015) and FUBAR (Murrell et al., 2013).

**Family**	**Number of recombination breakpoints**	**Number of positive selected sites**
Lipocalin	11	16
DAP36	6	7
Apyrase	3	1
Metalloprotease	3	0
Cystatin	1	0
Subolesin	1	0

## Data Availability Statement

The datasets presented in this study can be found in online repositories. The names of the repository/repositories and accession number(s) can be found in the article/**Supplementary Material**.

## Author Contributions

JR conceived the work and did the bioinformatic analysis. BM contributed in the data analysis and interpretation. All authors contributed to writing the manuscript.

## Conflict of Interest

The authors declare that the research was conducted in the absence of any commercial or financial relationships that could be construed as a potential conflict of interest.
