# Supplementary material for: TickSialoFam (TSFam): A Database That Helps to Classify Tick Salivary Proteins, a Review on Tick Salivary Protein Function and Evolution, With Considerations on the Tick Sialome Switching Phenomenon
Source: Front Cell Infect Microbiol. 2020 Jul 24;10:374. doi: 10.3389/fcimb.2020.00374 (PMC7396615; doi:10.3389/fcimb.2020.00374)
Supplement: Supplementary file 1 [file Data_Sheet_1.pdf]

Supplemental table 1: Functional classification of tick salivary secreted proteins following RPSBlast to the TickSialoFam database with a minimum e value of 1e-4 and minimum model coverage of 67%.

Group	Total	Argasidae	Prostriate	Amblyomminae	Haemaphysalinae	Hyalomminae	Rhipicephalinae	References
**Putative secreted**								
**Enzymes**								
5'nucleotidase	65	5	25	13	0	2	20	[1-7]
Ectonucleotide pyrophosphatase/phosphodiesterase	17	3	2	1	2	1	8	
IPPase	30	3	3	8	2	1	13	[8-12]#
Serine protease	135	4	19	32	12	1	67	
Longistatin	53	5	14	13	1	2	18	[13-15]
Metalloprotease	680	30	253	129	10	26	232	[16-22]
M13_peptidase	105	3	24	32	1	9	36	
Cysteinyl_peptidase	149	7	16	37	17	6	66	
Dipeptidyl peptidase	24	0	3	7	3	2	9	[23]#
Serine carboxypeptidase	135	4	19	32	12	1	67	
Zinc carboxypeptidase	10	0	3	3	1	0	3	
Endonuclease	90	9	18	29	10	1	23	
Coesterase	70	7	12	4	13	4	30	
Lipase	40	1	8	3	8	2	18	
Lipase, pancreatic type	0	0	0	0	0	0	0	
Phospholipase	40	3	12	7	6	2	10	[24-26]#
Sphingomyelinase	22	0	2	5	4	1	10	[27]#
Peroxidase	30	1	4	2	3	0	20	[28]
Superoxide dismutase	33	0	9	7	4	1	12	
**Protease inhibitors**								
Cystatin	185	9	20	54	9	2	91	[29-52]
Kazal	11	1	1	4	1	0	4	
Kunitz	1,882	41	921	426	19	25	450	[53-89]
Saposin	7	1	1	1	1	0	3	
Serpin	107	10	18	28	14	3	34	[90-111]
SPARC/Kazal	23	4	2	8	1		8	[112] *
Thyropin	50	0	5	16	2	0	27	[112]*
TIL	442	11	84	133	14	7	193	[113, 114]
Carboxypeptidase_inhibitor	61	1	1	12	0	1	46	[115]
**Tick -specific anti-clotting peptide**	21	0	0	21	0	0	0	
Madanin	17	0	0	1	0	6	10	
Tick Hirudin	21	0	0	21	0	0	0	[72, 116-119]
**Antimicrobial**								
Defensin	69	8	21	21	0	2	17	[120-132]
Lysozyme	21	2	1	8	2	0	8	[133]
Microplusin	148	8	25	55	7	5	48	[134]
DAE-2	18	1	1	5	3	1	7	[135]
5.3kDa	148	0	110	34	0	1	3	[136, 137]
**Immune related**								
Catalytically inactive chitinase-like lectin	65	9	11	8	7	2	28	
Complement receptor	14	3	4	2	1	0	4	
Complement-binding protein	7	1	3	1	1	0	1	
Ficolin/Ixoderin	78	4	39	4	9	1	21	[138]
Malectin	11	1	3	5	1	0	1	
HVA22/Cytokine	8	0	1	3	1	0	3	
Interleukin17-like	12	1	5	3	0	0	3	
ML_domain	115	14	35	33	5	4	24	[114]*
Niemann-Pick	20	0	4	5	0	0	11	
TGF-beta propeptide	32	2	5	3	2	3	17	
Peptidoglycan_recognition_protein	47	1	25	9	0	1	11	
Toll4_associated	17	0	2	9	1	0	5	
Toll-like	159	10	33	30	18	5	63	
**Ubiquitous protein families**								
Antigen-5	90	1	40	5	3	3	38	
Calcitonin/calcitonin-related, adrenomedullin-a	10	10	0	0	0	0	0	[139, 140]
CalreticulinCalnexin	28	3	3	12	1	1	8	[141-143]
Cell adhesion molecule	14	2	5	1	1	0	5	
Coiled-coil domain-containing	17	1	0	10	1	0	5	
CUTA1	5	0	0	3	0	0	2	
CystineKnotToxin	12	3	3	2	1	1	2	
Down syndrome cell adhesion molecule	103	14	15	13	10	3	48	
EFh_CREC_Calumenin_like	30	2	6	11	2	0	9	
Fasciclin-1	11	0	1	2	1	1	6	
Fukutin	7	0	2	0	0	0	5	
Hematopoietic stem/progenitor cells -like	14	0	3	6	1	0	4	
Insulin_growth_factor	68	1	11	15	6	3	32	[144, 145]
Integrin	22	2	3	3	2	0	0	
Kielin/chordin-like	12	1	3	2	1	1	4	
Laminin	34	3	5	3	3	0	20	
Lipocalin	3,695	186	893	1,175	124	122	1,195	[146-164]
Low-density lipoprotein receptor	61	10	13	9	3	3	23	
MAM-domain	10	0	7	1	1	0	1	
OSTMP1	7	1	3	1	0	0	2	
Phosphatidylethanolamine binding	17	1	2	7	1	0	6	

Selenoprotein	21	2	6	5	2	0	6	
Serum amyloid A	29	5	2	11	1	1	9	
Synaptotagmin 1	14	4	2	2	1	1	4	
TMEM9	10	0	1	6	0	0	3	
**Tick-specific families**								
Amb-25-357	5	0	0	5	0	0	0	
Mys-25-289	5	0	2	0	0	1	2	
10kDa-WC	433	0	433	0	0	0	0	
12kDa	74	4	7	50	5	1	7	
12kDaBasic	16	1	7	4	0	0	4	
13-14kDa	91	3	75	9	0	1	3	
13kDa-Basic	20	0	3	9	2	1	5	
15kDaBasic	24	0	0	24	0	0	0	
17.7kDa	11	11	0	0	0	0	0	
18kDa	65	0	12	33	3	4	13	
19kDa	77	0	0	63	2	0	12	
23-24kDa	150	6	33	39	14	7	51	
28kDa	117	0	0	18	1	3	95	
7DB	36	36	0	0	0	0	0	
8.9kDa	614	5	111	292	6	23	177	[114]*
8kDa	12	0	0	7	0	0	5	
AlaRich	97	4	18	37	4	4	30	[114]*
Basic Proline-rich	15	0	0	15	0	0	0	
BSMAP	11	0	1	4	2	0	4	
BTSP (Basic Tail)	926	51	498	129	11	25	212	[165-168]
Cytotoxin	242	14	103	63	11	1	50	[114]*
DAP-36	150	1	50	36	5	5	53	[169-171]
Derm9kDa	11	0	0	0	0	0	11	
Evasin	361	1	34	180	4	18	124	[172-179]
GRP	623	16	132	222	12	20	221	[114]*
Hya-40-323	14	0	0	0	0	14	0	
Hyp_94	45	0	0	45	0	0	0	
Hyp2009	32	0	0	19	0	0	13	
Hyp30	21	1	16	3	1	0	0	
Hyp669	3	0	0	2	0	0	1	
Iri-30-136	12	0	12	0	0	0	0	
Iric-25-199	5	0	5	0	0	0	0	
IRIC-25-235	4	0	4	0	0	0	0	
ISAC	172	0	172	0	0	0	0	[180-183]
Ixodegrin	476	1	252	102	3	9	109	[54, 184-186]
Ixodegrin-like	17	0	2	0	1	2	12	
Met-35-461	10	0	0	0	0	0	10	
Mucin	476	9	42	263	18	15	129	[114]*
MYS-2	6	0	2	1	1	1	1	
MYS-25-271	3	1	0	0	0	1	1	
Mys-25-299	6	0	1	1	0	0	4	
Mys-25-342	5	0	0	3	0	0	2	
MYS-3	2	1	1	0	0	0	0	
Mys-30-170	19	2	4	5	1	0	7	
Mys-30-60	53	0	14	32	0	0	7	[114]*
Mys-30-94	22	0	0	11	0	4	7	
MYS-5	8	0	4	1	0	2	1	
OneOfEach	146	0	9	47	2	4	84	
Papa	25	0	0	22	0	0	3	
Prich	120	1	28	64	1	1	25	
Rapp-25-325	6	0	0	0	0	0	6	
Rapp-40-287	18	0	0	0	0	0	18	
Rhi-30-197	16	0	0	0	0	1	15	
Rhi-40-385	8	0	0	0	0	0	8	
RHIP-25-139	13	0	0	0	0	1	12	
Rpul-30-84	19	0	0	0	0	0	19	
Salp15/Ixostatin	999	1	965	17	1	1	14	
Tick-MYS1	9	0	5	3	0	0	1	
TickMys-2	7	0	3	4	0	0	0	
Transposon	74	4	33	3	1	3	30	
Vitelogenin-VWF	23	4	1	0	2	3	13	
YRP	18	0	2	15	0	0	1	
**Total**	16,983	658	5,952	4,534	508	447	4,872	

# Enzymatic studies performed, but no demonstration of its molecular base.

* Indicates proteomic demonstration in saliva, but no functional study was completed.

References cited:

1. Ribeiro JM, Makoul GT, Levine J, Robinson DR, Spielman A: **Antihemostatic, antiinflammatory, and immunosuppressive properties of the saliva of a tick, Ixodes dammini**. *The Journal of experimental medicine* 1985, **161**(2):332-344.
2. Ribeiro JM, Endris TM, Endris R: **Saliva of the soft tick, *Ornithodoros moubata*, contains anti-platelet and apyrase activities**. *Comp Biochem Physiol A* 1991, **100**(1):109-112.
3. Willadsen P, Riding GA, Jarmey J, Atkins A: **The nucleotidase of *Boophilus microplus* and its relationship to enzymes from the rat and *Escherichia coli***. *Insect Biochem Molec Biol* 1993, **23**:291-295.
4. Mans BJ, Gaspar AR, Louw AI, Neitz AW: **Apyrase activity and platelet aggregation inhibitors in the tick *Ornithodoros savignyi* (Acari: Argasidae)**. *Experimental & applied acarology* 1998, **22**(6):353-366.

5. Mans BJ, Coetzee J, Louw AI, Gaspar AR, Neitz AW: **Disaggregation of aggregated platelets by apyrase from the tick, *Ornithodoros savignyi* (Acari: Argasidae)**. *Experimental & applied acarology* 2000, **24**(4):271-282.
6. Mans BJ, Andersen JF, Schwan TG, Ribeiro JM: **Characterization of anti-hemostatic factors in the argasid, *Argas monolakensis*: Implications for the evolution of blood-feeding in the soft tick family**. *Insect biochemistry and molecular biology* 2008, **38**(1):22-41.
7. Stutzer C, Mans BJ, Gaspar AR, Neitz AW, Maritz-Olivier C: ***Ornithodoros savignyi:* soft tick apyrase belongs to the 5'-nucleotidase family**. *Experimental parasitology* 2009, **122**(4):318-327.
8. Maritz-Olivier C, Louw AI, Neitz AWH: **Similar mechanisms regulate protein exocytosis from the salivary glands of ixodid and argasid ticks**. *Journal of Insect Physiology* 2005, **51**(12):1390-1396.
9. Yuan J, Bowman AS, Aljamali M, Payne MR, Tucker JS, Dillwith JW, Essenberg RC, Sauer JR: **Prostaglandin E-2-stimulated secretion of protein in the salivary glands of the lone star tick via a phosphoinositide signaling pathway**. *Insect biochemistry and molecular biology* 2000, **30**(11):1099-1106.
10. Qian Y, Yuan J, Essenberg RC, Bowman AS, Shook AL, Dillwith JW, Sauer JR: **Prostaglandin E2 in the salivary glands of the female tick, *Amblyomma americanum* (L.): calcium mobilization and exocytosis**. *Insect biochemistry and molecular biology* 1998, **28**(4):221-228.
11. McSwain JL, Tucker JS, Essemberg RC, Sauer JR: **Brain factor induced formation of inositol phosphates in tick salivary glands**. *Insect Biochem* 1989, **19**:343-349.
12. Essenberg RC, Mcswain JL, Roddy C, Sauer JR: **Agonist Induced Formation of Inositol Phosphates and Calcium Mobilization in Tick Salivary-Glands**. *Faseb Journal* 1988, **2**(5):A1374-A1374.
13. Anisuzzaman, Islam MK, Miyoshi T, Alim MA, Hatta T, Yamaji K, Matsumoto Y, Fujisaki K, Tsuji N: **Longistatin, a novel EF-hand protein from the ixodid tick Haemaphysalis longicornis, is required for acquisition of host blood-meals**. *International journal for parasitology* 2010, **40**(6):721-729.
14. Anisuzzaman, Khyrul Islam M, Abdul Alim M, Miyoshi T, Hatta T, Yamaji K, Matsumoto Y, Fujisaki K, Tsuji N: **Longistatin, a novel plasminogen activator from vector ticks, is resistant to plasminogen activator inhibitor-1**. *Biochemical and biophysical research communications* 2011, **413**(4):599-604.
15. Anisuzzaman, Islam MK, Alim MA, Miyoshi T, Hatta T, Yamaji K, Matsumoto Y, Fujisaki K, Tsuji N: **Longistatin, a Plasminogen Activator, Is Key to the Availability of Blood-Meals for Ixodid Ticks**. *Plos Pathogens* 2011, **7**(3).
16. Francischetti IM, Mather TN, Ribeiro JM: **Cloning of a salivary gland metalloprotease and characterization of gelatinase and fibrin(ogen)lytic activities in the saliva of the Lyme disease tick vector *Ixodes scapularis***. *Biochemical and biophysical research communications* 2003, **305**(4):869-875.
17. Francischetti IM, Mather TN, Ribeiro JM: **Tick saliva is a potent inhibitor of endothelial cell proliferation and angiogenesis**. *Thrombosis and haemostasis* 2005, **94**(1):167-174.
18. Harnnoi T, Sakaguchi T, Nishikawa Y, Xuan X, Fujisaki K: **Molecular characterization and comparative study of 6 salivary gland metalloproteases from the hard tick, *Haemaphysalis longicornis***. *Comparative biochemistry and physiology* 2007, **147**(1):93-101.
19. Decrem Y, Beaufays J, Blasioli V, Lahaye K, Brossard M, Vanhamme L, Godfroid E: **A family of putative metalloproteases in the salivary glands of the tick Ixodes ricinus**. *Febs Journal* 2008, **275**(7):1485-1499.
20. Imamura S, Vaz ID, Konnai S, Yamada S, Nakajima C, Onuma M, Ohashi K: **Effect of vaccination with a recombinant metalloprotease from Haemaphysalis longicornis**. *Experimental and Applied Acarology* 2009, **48**(4):345-358.
21. Ali A, Tirloni L, Isezaki M, Seixas A, Konnai S, Ohashi K, Vaz ID, Termignoni C: **Reprolysin metalloproteases from Ixodes persulcatus, Rhipicephalus sanguineus and Rhipicephalus microplus ticks**. *Experimental and Applied Acarology* 2014, **63**(4):559-578.
22. Ali A, Parizi LF, Guizzo MG, Tirloni L, Seixas A, Vaz ID, Termignoni C: **Immunoprotective potential of a Rhipicephalus (Boophilus) microplus metalloprotease**. *Veterinary parasitology* 2015, **207**(1-2):107-114.
23. Ribeiro JM, Mather TN: ***Ixodes scapularis*: salivary kininase activity is a metallo dipeptidyl carboxypeptidase**. *Experimental parasitology* 1998, **89**(2):213-221.
24. Dillwith JW, Surdick MR, Sauer JR, Essenberg RC: **Tick Salivary-Gland Phospholipase-A2**. *Faseb Journal* 1992, **6**(1):A495-A495.
25. Bowman AS, Gengler CL, Surdick MR, Zhu K, Essenberg RC, Sauer JR, Dillwith JW: **A novel phospholipase A(2) activity in saliva of the lone star tick, Amblyomma americanum (L.)**. *Experimental parasitology* 1997, **87**(2):121-132.
26. Zhu K, Bowman AS, Dillwith JW, Sauer JR: **Phospholipase A(2) activity in salivary glands and saliva of the lone star tick (Acari : Ixodidae) during tick feeding**. *Journal of medical entomology* 1998, **35**(4):500-504.
27. Alarcon-Chaidez FJ, Boppana VD, Hagymasi AT, Adler AJ, Wikel SK: **A novel sphingomyelinase-like enzyme in *Ixodes scapularis* tick saliva drives host CD4 T cells to express IL-4**. *Parasite immunology* 2009, **31**(4):210-219.
28. Tsuji N, Kamio T, Isobe T, Fujisaki K: **Molecular characterization of a peroxiredoxin from the hard tick Haemaphysalis longicornis**. *Insect molecular biology* 2001, **10**(2):121-129.
29. Kotsyfakis M, Sa-Nunes A, Francischetti IM, Mather TN, Andersen JF, Ribeiro JM: **Antiinflammatory and immunosuppressive activity of sialostatin L, a salivary cystatin from the tick *Ixodes scapularis***. *J Biol Chem* 2006, **281**(36):26298-26307.
30. Lima CA, Sasaki SD, Tanaka AS: **Bmcystatin, a cysteine proteinase inhibitor characterized from the tick *Boophilus microplus***. *Biochemical and biophysical research communications* 2006, **347**(1):44-50.
31. Grunclova L, Horn M, Vancova M, Sojka D, Franta Z, Mares M, Kopacek P: **Two secreted cystatins of the soft tick Ornithodoros moubata: differential expression pattern and inhibitory specificity**. *Biological chemistry* 2006, **387**(12):1635-1644.
32. Kotsyfakis M, Karim S, Andersen JF, Mather TN, Ribeiro JM: **Selective cysteine protease inhibition contributes to blood-feeding success of the tick *Ixodes scapularis***. *The Journal of biological chemistry* 2007, **282**(40):29256-29263.
33. Kotsyfakis M, Anderson JM, Andersen JF, Calvo E, Francischetti IM, Mather TN, Valenzuela JG, Ribeiro JM: **Cutting edge: Immunity against a "silent" salivary antigen of the Lyme vector *Ixodes scapularis* impairs its ability to feed**. *J Immunol* 2008, **181**(8):5209-5212.
34. Yamaji K, Tsuji N, Miyoshi T, Islam MK, Hatta T, Alim MA, Anisuzzaman M, Kushibiki S, Fujisaki K: **A salivary cystatin, HlSC-1, from the ixodid tick Haemaphysalis longicornis play roles in the blood-feeding processes**. *Parasitology research* 2009, **106**(1):61-68.
35. Kotsyfakis M, Horka H, Salat J, Andersen JF: **The crystal structures of two salivary cystatins from the tick Ixodes scapularis and the effect of these inhibitors on the establishment of Borrelia burgdorferi infection in a murine model**. *Molecular microbiology* 2010, **77**(2):456-470.
36. Salat J, Paesen GC, Rezacova P, Kotsyfakis M, Kovarova Z, Sanda M, Majtan J, Grunclova L, Horka H, Andersen JF *et al*: **Crystal structure and functional characterization of an immunomodulatory salivary cystatin from the soft tick Ornithodoros moubata**. *Biochemical Journal* 2010, **429**:103-112.
37. Ibelli AM, Hermance MM, Kim TK, Gonzalez CL, Mulenga A: **Bioinformatics and expression analyses of the Ixodes scapularis tick cystatin family**. *Experimental & applied acarology* 2012.
38. Lieskovska J, Sirmarova J, Kotsyfakis M, Kopecky J: **A novel mechanism of action in dendritic cells for a tick salivary immunomodulatory cystatin**. *Immunology* 2012, **137**:246-246.
39. Schwarz A, Valdes JJ, Kotsyfakis M: **The role of cystatins in tick physiology and blood feeding**. *Ticks and tick-borne diseases* 2012, **3**(3):117-127.
40. Parizi LF, Githaka NW, Acevedo C, Benavides U, Seixas A, Logullo C, Konnai S, Ohashi K, Masuda A, Vaz ID: **Sequence characterization and immunogenicity of cystatins from the cattle tick Rhipicephalus (Boophilus) microplus**. *Ticks and tick-borne diseases* 2013, **4**(6):492-499.
41. Chen G, Wang X, Severo MS, Sakhon OS, Sohail M, Brown LJ, Sircar M, Snyder GA, Sundberg EJ, Ulland TK *et al*: **The tick salivary protein sialostatin L2 inhibits caspase-1-mediated inflammation during *Anaplasma phagocytophilum* infection**. *Infect Immun* 2014, **82**(6):2553-2564. doi: 2510.1128/IAI.01679-01614. Epub 02014 Mar 01631.
42. Lieskovska J, Palenikova J, Langhansova H, Campos Chagas A, Calvo E, Kotsyfakis M, Kopecky J: **Tick sialostatins L and L2 differentially influence dendritic cell responses to *Borrelia* spirochetes**. *Parasit Vectors* 2015, **8:275.**(doi):10.1186/s13071-13015-10887-13071.
43. Klein M, Bruhl TJ, Staudt V, Reuter S, Grebe N, Gerlitzki B, Hoffmann M, Bohn T, Ulges A, Stergiou N *et al*: **Tick Salivary Sialostatin L Represses the Initiation of Immune Responses by Targeting IRF4-Dependent Transcription in Murine Mast Cells**. *J Immunol* 2015, **195**(2):621-631. doi: 610.4049/jimmunol.1401823. Epub 1402015 Jun 1401815.
44. Lieskovska J, Palenikova J, Sirmarova J, Elsterova J, Kotsyfakis M, Campos Chagas A, Calvo E, Ruzek D, Kopecky J: **Tick salivary cystatin sialostatin L2 suppresses IFN responses in mouse dendritic cells**. *Parasite immunology* 2015, **37**(2):70-78.
45. Wang YJ, Yu XM, Cao J, Zhou YZ, Gong HY, Zhang HS, Li XR, Zhou JL: **Characterization of a secreted cystatin from the tick Rhipicephalus haemaphysaloides**. *Experimental and Applied Acarology* 2015, **67**(2):289-298.
46. Parizi LF, Sabadin GA, Alzugaray MF, Seixas A, Logullo C, Konnai S, Ohashi K, Masuda A, Vaz ID: **Rhipicephalus microplus and Ixodes ovatus cystatins in tick blood digestion and evasion of host immune response**. *Parasite Vector* 2015, **8**.
47. Zavasnik-Bergant T, Vidmar R, Sekirnik A, Fonovic M, Salat J, Grunclova L, Kopacek P, Turk B: **Salivary Tick Cystatin OmC2 Targets Lysosomal Cathepsins S and C in Human Dendritic Cells**. *Frontiers in cellular and infection microbiology* 2017, **7**.
48. Rangel CK, Parizi LF, Sabadin GA, Costa EP, Romeiro NC, Isezaki M, Githaka NW, Seixas A, Logullo C, Konnai S *et al*: **Molecular and structural characterization of novel cystatins from the taiga tick Ixodes persulcatus**. *Ticks and tick-borne diseases* 2017, **8**(3):432-441.
49. Rahman MK, Islam MS, You M: **Impact of Subolesin and Cystatin Knockdown by RNA Interference in Adult Female Haemaphysalis longicornis (Acari: Ixodidae) on Blood Engorgement and Reproduction**. *Insects* 2018, **9**(2).
50. Sun T, Wang FQ, Pan W, Wu QH, Wang JW, Dai JF: **An Immunosuppressive Tick Salivary Gland Protein DsCystatin Interferes With Toll-Like Receptor Signaling by Downregulating TRAF6**. *Frontiers in immunology* 2018, **9**.
51. Kotal J, Stergiou N, Busa M, Chlastakova A, Berankova Z, Rezacova P, Langhansova H, Schwarz A, Calvo E, Kopecky J *et al*: **The structure and function of Iristatin, a novel immunosuppressive tick salivary cystatin**. *Cellular and Molecular Life Sciences* 2019, **76**(10):2003-2013.
52. Wei NN, Lin ZB, Xu ZM, Gong HY, Zhang HS, Zhou YS, Cao J, Li GQ, Zhou JL: **Immunosuppressive effects of tick protein RHcyst-1 on murine bone marrow-derived dendritic cells**. *Parasite Vector* 2019, **12**.
53. Francischetti IM, Valenzuela JG, Andersen JF, Mather TN, Ribeiro JM: **Ixolaris, a novel recombinant tissue factor pathway inhibitor (TFPI) from the salivary gland of the tick, *Ixodes scapularis*: identification of factor X and factor Xa as scaffolds for the inhibition of factor VIIa/tissue factor complex**. *Blood* 2002, **99**(10):3602-3612.
54. Mans BJ, Louw AI, Neitz AW: **Savignygrin, a platelet aggregation inhibitor from the soft tick *Ornithodoros savignyi*, presents the RGD integrin recognition motif on the Kunitz-BPTI fold**. *The Journal of biological chemistry* 2002, **277**(24):21371-21378.
55. Mans BJ, Louw AI, Neitz AWH: **Amino acid sequence and structure modeling of savignin, a thrombin inhibitor from the tick, Ornithodoros savignyi**. *Insect biochemistry and molecular biology* 2002, **32**(7):821-828.
56. Mans BJ, Louw AI, Neitz AWH: **Savignygrin, a platelet aggregation inhibitor from the soft tick Ornithodoros savignyi, presents the RGD integrin recognition motif on the Kunitz-BPTI fold**. *Journal of Biological Chemistry* 2002, **277**(24):21371-21378.
57. Francischetti IM, Mather TN, Ribeiro JM: **Penthalaris, a novel recombinant five-Kunitz tissue factor pathway inhibitor (TFPI) from the salivary gland of the tick vector of Lyme disease, *Ixodes scapularis***. *Thrombosis and haemostasis* 2004, **91**(5):886-898.
58. Lai R, Takeuchi H, Jonczy J, Rees HH, Turner PC: **A thrombin inhibitor from the ixodid tick, *Amblyomma hebraeum***. *Gene* 2004, **342**(2):243-249.
59. Monteiro RQ, Rezaie AR, Ribeiro JM, Francischetti IM: **Ixolaris: a factor Xa heparin-binding exosite inhibitor**. *The Biochemical journal* 2005, **387**(Pt 3):871-877.
60. Kato N, Okayama T, Isawa H, Yuda M, Chinzei Y, Iwanaga S: **Contribution of the N-terminal and C-terminal domains of haemaphysalin to inhibition of activation of plasma kallikrein-kinin system**. *Journal of biochemistry* 2005, **138**(3):225-235.
61. Kato N, Iwanaga S, Okayama T, Isawa H, Yuda M, Chinzei Y: **Identification and characterization of the plasma kallikrein-kinin system inhibitor, haemaphysalin, from hard tick, Hoemaphysalis longicornis**. *Thrombosis and haemostasis* 2005, **93**(2):359-367.
62. Nazareth RA, Tomaz LS, Ortiz-Costa S, Atella GC, Ribeiro JM, Francischetti IM, Monteiro RQ: **Antithrombotic properties of Ixolaris, a potent inhibitor of the extrinsic pathway of the coagulation cascade**. *Thrombosis and haemostasis* 2006, **96**(1):7-13.
63. Sasaki SD, Cotrin SS, Carmona AK, Tanaka AS: **An unexpected inhibitory activity of Kunitz-type serine proteinase inhibitor derived from Boophilus microplus trypsin inhibitor on cathepsin L**. *Biochemical and biophysical research communications* 2006, **341**(1):266-272.
64. Paesen GC, Siebold C, Harlos K, Peacey MF, Nuttall PA, Stuart DI: **A tick protein with a modified Kunitz fold inhibits human tryptase**. *Journal of molecular biology* 2007, **368**(4):1172-1186.
65. Monteiro RQ, Rezaie AR, Bae JS, Calvo E, Andersen JF, Francischetti IM: **Ixolaris binding to factor X reveals a precursor state of factor Xa heparin-binding exosite**. *Protein Sci* 2008, **17**(1):146-153.
66. Sasaki SD, Tanaka AS: **rBmTI-6, a Kunitz-BPTI domain protease inhibitor from the tick Boophilus microplus, its cloning, expression and biochemical characterization**. *Veterinary parasitology* 2008, **155**(1-2):133-141.
67. Macedo-Ribeiro S, Almeida C, Calisto BM, Friedrich T, Mentele R, Sturzebecher J, Fuentes-Prior P, Pereira PJB: **Isolation, Cloning and Structural Characterisation of Boophilin, a Multifunctional Kunitz-Type Proteinase Inhibitor from the Cattle Tick**. *PLoS ONE* 2008, **3**(2).
68. Monteiro RQ, Rezaie AR, Bae JS, Calvo E, Andersen JF, Francischetti IMB: **Ixolaris binding to factor X reveals a precursor state of factor Xa heparin-binding exosite**. *Protein Science* 2008, **17**(1):146-153.
69. Islam MK, Tsuji N, Miyoshi T, Alim MA, Huang X, Hatta T, Fujisaki K: **The Kunitz-like modulatory protein haemangin is vital for hard tick blood-feeding success**. *PLoS Pathog* 2009, **5**(7):e1000497.
70. Decrem Y, Rath G, Blasioli V, Cauchie P, Robert S, Beaufays J, Frere JM, Feron O, Dogne JM, Dessy C *et al*: **Ir-CPI, a coagulation contact phase inhibitor from the tick Ixodes ricinus, inhibits thrombus formation without impairing hemostasis**. *Journal of Experimental Medicine* 2009, **206**(11):2381-2395.
71. Paesen GC, Siebold C, Dallas ML, Peers C, Harlos K, Nuttall PA, Nunn MA, Stuart DI, Esnouf RM: **An Ion-channel Modulator from the Saliva of the Brown Ear Tick has a Highly Modified Kunitz/BPTI Structure**. *Journal of molecular biology* 2009, **389**(4):734-747.
72. Liao M, Zhou JL, Gong HY, Boldbaatar D, Shirafuji R, Battur B, Nishikawa Y, Fujisaki K: **Hemalin, a thrombin inhibitor isolated from a midgut cDNA library from the hard tick Haemaphysalis longicornis**. *Journal of Insect Physiology* 2009, **55**(2):164-173.
73. Lima CA, Torquato RJS, Sasaki SD, Justo GZ, Tanaka AS: **Biochemical characterization of a Kunitz type inhibitor similar to dendrotoxins produced by Rhipicephalus (Boophilus) microplus (Acari: Ixodidae) hemocytes**. *Veterinary parasitology* 2010, **167**(2-4):279-287.
74. Batista IFC, Ramos OHP, Ventura JS, Junqueira-De-Azevedo ILM, Ho PL, Chudzinski-Tavassi AM: **A new Factor Xa inhibitor from Amblyomma cajennense with a unique domain composition**. *Archives of biochemistry and biophysics* 2010, **493**(2):151-156.
75. Gao XA, Shi L, Zhou YZ, Cao J, Zhang HS, Zhou JL: **Characterization of the anticoagulant protein Rhipilin-1 from the Rhipicephalus haemaphysaloides tick**. *Journal of Insect Physiology* 2011, **57**(2):339-343.
76. Andreotti R, Cunha RC, Soares MA, Guerrero FD, Leite FPL, de Leon AAP: **Protective immunity against tick infestation in cattle vaccinated with recombinant trypsin inhibitor of Rhipicephalus microplus**. *Vaccine* 2012, **30**(47):6678-6685.
77. Akagi EM, de Sa PL, Simons SM, Bellini MH, Barreto SA, Chudzinski-Tavassi AM: **Pro-apoptotic effects of Amblyomin-X in murine renal cell carcinoma "in vitro"**. *Biomed Pharmacother* 2012, **66**(1):64-69.
78. Dai SX, Zhang AD, Huang JF: **Evolution, expansion and expression of the Kunitz/BPTI gene family associated with long-term blood feeding in Ixodes Scapularis**. *BMC evolutionary biology* 2012, **12**.
79. Valdes JJ, Schwarz A, Cabeza de Vaca I, Calvo E, Pedra JH, Guallar V, Kotsyfakis M: **Tryptogalinin is a tick Kunitz serine protease inhibitor with a unique intrinsic disorder**. *PLoS ONE* 2013, **8**(5):e62562.
80. Cao J, Shi L, Zhou YZ, Gao X, Zhang HS, Gong HY, Zhou JL: **CHARACTERIZATION OF A NEW KUNITZ-TYPE SERINE PROTEASE INHIBITOR FROM THE HARD TICK Rhipicephalus hemaphysaloides**. *Archives of insect biochemistry and physiology* 2013, **84**(2):104-113.
81. Liu XY, de la Fuente J, Cote M, Galindo RC, Moutailler S, Vayssier-Taussat M, Bonnet SI: **IrSPI, a Tick Serine Protease Inhibitor Involved in Tick Feeding and Bartonella henselae Infection**. *PLoS neglected tropical diseases* 2014, **8**(7).
82. Pasqualoto KFM, Balan A, Barreto SA, Simons SM, Chudzinski-Tavassi AM: **Structural Findings and Molecular Modeling Approach of a TFPI-Like Inhibitor**. *Protein and peptide letters* 2014, **21**(5):452-457.
83. Morais KLP, Pasqualoto KFM, Pacheco MTF, Berra CM, Alvarez-Flores MP, Chudzinski-Tavassi AM: **Rational development of a novel TFPI-like inhibitor from Amblyomma cajennense tick**. *Toxin Rev* 2014, **33**(1-2):48-52.
84. Valdes JJ, Moal IH: **Prediction of Kunitz ion channel effectors and protease inhibitors from the Ixodes ricinus sialome**. *Ticks and tick-borne diseases* 2014, **5**(6):947-950.
85. Branco VG, Iqbal A, Alvarez-Flores MP, Sciani JM, de Andrade SA, Iwai LK, Serrano SMT, Chudzinski-Tavassi AM: **Amblyomin-X having a Kunitz-type homologous domain, is a noncompetitive inhibitor of FXa and induces anticoagulation in vitro and in vivo**. *Bba-Proteins Proteom* 2016, **1864**(10):1428-1435.
86. de Souza JG, Morais KLP, Angles-Cano E, Boufleur P, de Mello ES, Maria DA, Origassa CST, Zampolli HD, Camara NOS, Berra CM *et al*: **Promising pharmacological profile of a Kunitz-type inhibitor in murine renal cell carcinoma model**. *Oncotarget* 2016, **7**(38):62255-62266.
87. Soares TS, Oliveira F, Torquato RJS, Sasaki SD, Araujo MS, Paschoalin T, Tanaka AS: **BmTI-A, a Kunitz type inhibitor from Rhipicephalus microplus able to interfere in vessel formation**. *Veterinary parasitology* 2016, **219**:44-52.
88. De Paula VS, Silva FHS, Francischetti IMB, Monteiro RQ, Valente AP: **H-1, N-15 and C-13 resonance assignments of Ixolaris, a tissue factor pathway inhibitor from the tick salivary gland**. *Biomol Nmr Assign* 2017, **11**(2):293-296.
89. Zhang HS, Qiao RQ, Gong HY, Cao J, Zhou YZ, Zhou JL: **Identification and anticoagulant activity of a novel Kunitz-type protein HA11 from the salivary gland of the tick Hyalomma asiaticum**. *Experimental and Applied Acarology* 2017, **71**(1):71-85.
90. Mulenga A, Sugino M, Nakajima M, Sugimoto C, Onuma M: **Tick-encoded serine proteinase inhibitors (Serpins); Potential target antigens for tick vaccine development**. *Journal of Veterinary Medical Science* 2001, **63**(10):1063-1069.
91. Sugino M, Imamura S, Mulenga A, Nakajima M, Tsuda A, Ohashi K, Onuma M: **A serine proteinase inhibitor (serpin) from ixodid tick Haemaphysalis longicornis; cloning and preliminary assessment of its suitability as a candidate for a tick vaccine**. *Vaccine* 2003, **21**(21-22):2844-2851.
92. Mulenga A, Tsuda A, Onuma M, Sugimoto C: **Four serine proteinase inhibitors (serpin) from the brown ear tick, Rhiphicephalus appendiculatus; cDNA cloning and preliminary characterization**. *Insect biochemistry and molecular biology* 2003, **33**(2):267-276.
93. Imamura S, da Silva Vaz Junior I, Sugino M, Ohashi K, Onuma M: **A serine protease inhibitor (serpin) from Haemaphysalis longicornis as an anti-tick vaccine**. *Vaccine* 2005, **23**(10):1301-1311.
94. Prevot PP, Adam B, Boudjeltia KZ, Brossard M, Lins L, Cauchie P, Brasseur R, Vanhaeverbeek M, Vanhamme L, Godfroid E: **Anti-hemostatic effects of a serpin from the saliva of the tick Ixodes ricinus**. *The Journal of biological chemistry* 2006, **281**(36):26361-26369.
95. Prevot PP, Couvreur B, Denis V, Brossard M, Vanhamme L, Godfroid E: **Protective immunity against *Ixodes ricinus* induced by a salivary serpin**. *Vaccine* 2007, **25**(17):3284-3292.
96. Mulenga A, Khumthong R, Blandon MA: **Molecular and expression analysis of a family of the Amblyomma americanum tick Lospins**. *Journal of Experimental Biology* 2007, **210**(18):3188-3198.
97. Mulenga A, Khumthong R, Chalaire KC: **Ixodes scapularis tick serine proteinase inhibitor (serpin) gene family; annotation and transcriptional analysis**. *BMC genomics* 2009, **10**.
98. Gao JL, Luo JX, Fan RQ, Schulte-Spechtel UC, Fingerle V, Guan GQ, Zhao HP, Li YQ, Ren QY, Ma ML *et al*: **Characterization of a concealed antigen Hq05 from the hard tick Haemaphysalis qinghaiensis and its effect as a vaccine against tick infestation in sheep**. *Vaccine* 2009, **27**(3):483-490.
99. Kovarova Z, Chmelar J, Sanda M, Brynda J, Mares M, Rezacova P: **Crystallization and diffraction analysis of the serpin IRS-2 from the hard tick Ixodes ricinus**. *Acta Crystallogr F* 2010, **66**:1453-1457.
100. Jittapalapong S, Kaewhom P, Pumhom P, Canales M, de la Fuente J, Stich RW: **Immunization of rabbits with recombinant serine protease inhibitor reduces the performance of adult female Rhipicephalus microplus**. *Transboundary and emerging diseases* 2010, **57**(1-2):103-106.
101. Chalaire KC, Kim TK, Garcia-Rodriguez H, Mulenga A: **Amblyomma americanum (L.) (Acari: Ixodidae) tick salivary gland serine protease inhibitor (serpin) 6 is secreted into tick saliva during tick feeding**. *Journal of Experimental Biology* 2011, **214**(4):665-673.
102. Rodriguez-Valle M, Vance M, Moolhuijzen PM, Tao X, Lew-Tabor AE: **Differential recognition by tick-resistant cattle of the recombinantly expressed Rhipicephalus microplus serine protease inhibitor-3 (RMS-3)**. *Ticks and tick-borne diseases* 2012, **3**(3):159-169.
103. Yu YF, Cao J, Zhou YZ, Zhang HS, Zhou JL: **Isolation and characterization of two novel serpins from the tick Rhipicephalus haemaphysaloides**. *Ticks and tick-borne diseases* 2013, **4**(4):297-303.
104. Tirloni L, Seixas A, Mulenga A, Vaz ID, Termignoni C: **A family of serine protease inhibitors (serpins) in the cattle tick Rhipicephalus (Boophilus) microplus**. *Experimental parasitology* 2014, **137**:25-34.
105. Kim TK, Tirloni L, Radulovic Z, Lewis L, Bakshi M, Hill C, Vaz ID, Logullo C, Termignoni C, Mulenga A: **Conserved Amblyomma americanum tick Serpin19, an inhibitor of blood clotting factors Xa and XIa, trypsin and plasmin, has anti-haemostatic functions**. *International journal for parasitology* 2015, **45**(9-10):613-627.
106. Palenkova J, Lieskovska J, Langhansova H, Kotsyfakis M, Chmelar J, Kopecky J: **Ixodes ricinus Salivary Serpin IRS-2 Affects Th17 Differentiation via Inhibition of the Interleukin-6/STAT-3 Signaling Pathway**. *Infection and immunity* 2015, **83**(5):1949-1956.
107. Porter L, Radulovic Z, Kim T, Braz GRC, Vaz ID, Mulenga A: **Bioinformatic analyses of male and female Amblyomma americanum tick expressed serine protease inhibitors (serpins)**. *Ticks and tick-borne diseases* 2015, **6**(1):16-30.
108. Tirloni L, Kim TK, Coutinho ML, Ali A, Seixas A, Termignoni C, Mulenga A, Vaz ID: **The putative role of Rhipicephalus microplus salivary serpins in the tick-host relationship**. *Insect biochemistry and molecular biology* 2016, **71**:12-28.
109. Toyomane K, Konnai S, Niwa A, Githaka N, Isezaki M, Yamada S, Ito T, Takano A, Ando S, Kawabata H *et al*: **Identification and the preliminary in vitro characterization of IRIS homologue from salivary glands of Ixodes persulcatus Schulze**. *Ticks and tick-borne diseases* 2016, **7**(1):119-125.
110. Chmelar J, Kotal J, Langhansova H, Kotsyfakis M: **Protease Inhibitors in Tick Saliva: The Role of Serpins and Cystatins in Tick-host-Pathogen Interaction**. *Frontiers in cellular and infection microbiology* 2017, **7**.
111. Xu ZM, Lin ZB, Wei NN, Di Q, Cao J, Zhou YZ, Gong HY, Zhang HS, Zhou JL: **Immunomodulatory effects of Rhipicephalus haemaphysaloides serpin RHS2 on host immune responses**. *Parasite Vector* 2019, **12**.

112. Oliveira CJ, Anatriello E, de Miranda-Santos IK, Francischetti IM, Sa-Nunes A, Ferreira BR, Ribeiro JM: **Proteome of *Rhipicephalus sanguineus* tick saliva induced by the secretagogues pilocarpine and dopamine**. *Ticks and tick-borne diseases* 2013, **4**(6):469-477.
113. Sasaki SD, de Lima CA, Lovato DV, Juliano MA, Torquato RJ, Tanaka AS: **BmSI-7, a novel subtilisin inhibitor from *Boophilus microplus*, with activity toward Pr1 proteases from the fungus *Metarhizium anisopliae***. *Experimental parasitology* 2008, **118**(2):214-220.
114. Kim TK, Tirloni L, Pinto AFM, Moresco J, Yates JR, Vaz ID, Mulenga A: ***Ixodes scapularis* tick saliva proteins sequentially secreted every 24 h during blood feeding**. *PLoS neglected tropical diseases* 2016, **10**(1).
115. Arolas JL, Lorenzo J, Rovira A, Castella J, Aviles FX, Sommerhoff CP: **A carboxypeptidase inhibitor from the tick *Rhipicephalus bursa*: isolation, cDNA cloning, recombinant expression, and characterization**. *The Journal of biological chemistry* 2005, **280**(5):3441-3448.
116. Koh CY, Kazimirova M, Trimnell A, Takac P, Labuda M, Nuttall PA, Kini RM: **Variegin, a novel fast and tight binding thrombin inhibitor from the tropical bont tick**. *Journal of Biological Chemistry* 2007, **282**(40):29101-29113.
117. Koh CY, Kumar S, Kazimirova M, Nuttall PA, Radhakrishnan UP, Kim S, Jagadeeswaran P, Imamura T, Mizuguchi J, Iwanaga S *et al*: **Crystal structure of thrombin in complex with S-variegin: insights of a novel mechanism of inhibition and design of tunable thrombin inhibitors**. *PLoS ONE* 2011, **6**(10):e26367.
118. Iyer JK, Koh CY, Kazimirova M, Roller L, Jobichen C, Swaminathan K, Mizuguchi J, Iwanaga S, Nuttall PA, Chan MY *et al*: **Avathrin: a novel thrombin inhibitor derived from a multicopy precursor in the salivary glands of the ixodid tick, Amblyomma variegatum**. *Faseb Journal* 2017, **31**(7):2981-2995.
119. Iqbal A, Goldfeder MB, Marques-Porto R, Asif H, de Souza JG, Faria F, Chudzinski-Tavassi AM: **Revisiting antithrombotic therapeutics; sculptin, a novel specific, competitive, reversible, scissile and tight binding inhibitor of thrombin**. *Scientific reports* 2017, **7**.
120. Zhou J, Liao M, Ueda M, Gong H, Xuan X, Fujisaki K: **Sequence characterization and expression patterns of two defensin-like antimicrobial peptides from the tick *Haemaphysalis longicornis***. *Peptides* 2007, **28**(6):1304-1310.
121. Todd SM, Sonenshine DE, Hynes WL: **Tissue and life-stage distribution of a defensin gene in the Lone Star tick, Amblyomma americanum**. *Medical and veterinary entomology* 2007, **21**(2):141-147.
122. Kocan KM, de La Fuente J, Manzano-Roman R, Naranjo V, Hynes WL, Sonenshine DE: **Silencing expression of the defensin, varisin, in male Dermacentor variabilis by RNA interference results in reduced Anaplasma marginale infections**. *Experimental and Applied Acarology* 2008, **46**(1-4):17-28.
123. Liu ZG, Liu H, Liu XY, Wu XL: **Purification and cloning of a novel antimicrobial peptide from salivary glands of the hard tick, Ixodes sinensis**. *Comp Biochem Phys B* 2008, **149**(4):557-561.
124. Saito Y, Konnai S, Yamada S, Imamura S, Nishikado H, Ito T, Onuma M, Ohashi K: **Identification and characterization of antimicrobial peptide, defensin, in the taiga tick, Ixodes persulcatus**. *Insect molecular biology* 2009, **18**(4):531-539.
125. Lu XY, Chen QL, Lv Y, Wang MJ, Lu ZK, Feng FF, Liu JZ, Yu HN: **A novel defensin-like peptide from salivary glands of the hard tick, Haemaphysalis longicornis**. *Protein Science* 2010, **19**(3):392-397.
126. Zhang HS, Zhang WJ, Wang XZ, Zhou YZ, Wang N, Zhou JL: **Identification of a cysteine-rich antimicrobial peptide from salivary glands of the tick Rhipicephalus haemaphysaloides**. *Peptides* 2011, **32**(3):441-446.
127. Rathore B, Faridi F, Bargujar J, Ghorui SK, Manohar GS: **Isolation of Defensin Gene from Salivary Gland of Hyalomma Dromedarii Ticks from Camelus Dromedarius by Polymerase Chain Reaction**. *J Camel Pract Res* 2013, **20**(1):125-127.
128. Tonk M, Cabezas-Cruz A, Valdes JJ, Rego RO, Rudenko N, Golovchenko M, Bell-Sakyi L, de la Fuente J, Grubhoffer L: **Identification and partial characterisation of new members of the Ixodes ricinus defensin family**. *Gene* 2014, **540**(2):146-152.
129. Tonk M, Cabezas-Cruz A, Valdes JJ, Rego ROM, Chrudimska T, Strnad M, Sima R, Bell-Sakyi L, Franta Z, Vilcinskas A *et al*: **Defensins from the tick Ixodes scapularis are effective against phytopathogenic fungi and the human bacterial pathogen Listeria grayi**. *Parasite Vector* 2014, **7**.
130. Chrudimska T, Cerovsky V, Slaninova J, Rego ROM, Grubhoffer L: **Defensin from the ornate sheep tick Dermacentor marginatus and its effect on Lyme borreliosis spirochetes**. *Developmental and comparative immunology* 2014, **46**(2):165-170.
131. Tonk M, Cabezas-Cruz A, Valdes JJ, Rego ROM, Rudenko N, Golovchenko M, Bell-Sakyi L, de la Fuente J, Grubhoffer L: **Identification and partial characterisation of new members of the Ixodes ricinus defensin family**. *Gene* 2014, **540**(2):146-152.
132. Wang JJ, Bian G, Pan W, Feng TT, Dai JF: **Molecular characterization of a defensin gene from a hard tick, Dermacentor silvarum**. *Parasite Vector* 2015, **8**.
133. Tanaka T, Kawano S, Nakao S, Umemiya-Shirafuji R, Rahman MM, Boldbaatar D, Battur B, Liao M, Fujisaki K: **The identification and characterization of lysozyme from the hard tick Haemaphysalis longicornis**. *Ticks and tick-borne diseases* 2010, **1**(4):178-185.
134. Fogaca AC, Lorenzini DM, Kaku LM, Esteves E, Bulet P, Daffre S: **Cysteine-rich antimicrobial peptides of the cattle tick Boophilus microplus: isolation, structural characterization and tissue expression profile**. *Developmental and comparative immunology* 2004, **28**(3):191-200.
135. Chou S, Daugherty MD, Peterson SB, Biboy J, Yang YY, Jutras BL, Fritz-Laylin LK, Ferrin MA, Harding BN, Jacobs-Wagner C *et al*: **Transferred interbacterial antagonism genes augment eukaryotic innate immune function**. *Nature* 2015, **518**(7537):98-+.
136. Pichu S, Ribeiro JM, Mather TN: **Purification and characterization of a novel salivary antimicrobial peptide from the tick, *Ixodes scapularis***. *Biochemical and biophysical research communications* 2009, **390**(3):511-515.
137. Liu L, Dai J, Zhao YO, Narasimhan S, Yang Y, Zhang L, Fikrig E: **Ixodes scapularis JAK-STAT pathway regulates tick antimicrobial peptides, thereby controlling the agent of human granulocytic anaplasmosis**. *The Journal of infectious diseases* 2012, **206**(8):1233-1241.
138. Rego RO, Hajdusek O, Kovar V, Kopacek P, Grubhoffer L, Hypsa V: **Molecular cloning and comparative analysis of fibrinogen-related proteins from the soft tick *Ornithodoros moubata* and the hard tick *Ixodes ricinus***. *Insect biochemistry and molecular biology* 2005, **35**(9):991-1004.
139. Iwanaga S, Isawa H, Yuda M: **Horizontal gene transfer of a vertebrate vasodilatory hormone into ticks**. *Nature communications* 2014, **5**.
140. Francischetti IMB, Meng ZJ, Mans BJ, Gudderra N, Hall M, Veenstra TD, Pham VM, Kotsyfakis M, Ribeiro JMC: **An insight into the salivary transcriptome and proteome of the soft tick and vector of epizootic bovine abortion, Ornithodoros coriaceus**. *Journal of proteomics* 2008, **71**(5):493-512.
141. Hai VV, Pages F, Boulanger N, Audebert S, Parola P, Almeras L: **Immunoproteomic identification of antigenic salivary biomarkers detected by Ixodes ricinus-ex posed rabbit sera**. *Ticks and tick-borne diseases* 2013, **4**(5):459-468.
142. Sanders ML, Jaworski DC, Sanchez JL, DeFraites RE, Glass GE, Scott AL, Raha S, Ritchie BC, Needham GR, Schwartz BS: **Antibody to a cDNA-derived calreticulin protein from Amblyomma americanum as a biomarker of tick exposure in humans**. *American Journal of Tropical Medicine and Hygiene* 1998, **59**(2):279-285.
143. Jaworski DCSFA, Lamoreaux W, Coons LB, Muller MT, Needham GR: **A secreted calreticulin protein in ixodid tick (*Amblyomma americanum*) saliva**. *J Insect Physiol* 1995, **41**:369-375.
144. Radulovic ZM, Porter LM, Kim TK, Bakshi M, Mulenga A: **Amblyomma americanum tick saliva insulin-like growth factor binding protein-related protein 1 binds insulin but not insulin-like growth factors**. *Insect molecular biology* 2015, **24**(5):539-550.
145. Mulenga A, Khumthong R: **Silencing of three Amblyomma americanum (L.) insulin-like growth factor binding protein-related proteins prevents ticks from feeding to repletion**. *Journal of Experimental Biology* 2010, **213**(7):1153-1161.
146. Paesen GC, Adams PL, Harlos K, Nuttall PA, Stuart DI: **Tick histamine-binding proteins: Isolation, cloning, and three-dimensional structure**. *Molecular cell* 1999, **3**(5):661-671.
147. Sangamnatdej S, Paesen GC, Slovak M, Nuttall PA: **A high affinity serotonin- and histamine-binding lipocalin from tick saliva**. *Insect molecular biology* 2002, **11**(1):79-86.
148. Mans BJ, Louw AI, Neitz AW: **The major tick salivary gland proteins and toxins from the soft tick, *Ornithodoros savignyi*, are part of the tick Lipocalin family: implications for the origins of tick toxicoses**. *Mol Biol Evol* 2003, **20**(7):1158-1167.
149. Mans BJ: **Tick histamine-binding proteins and related lipocalins: potential as therapeutic agents**. *Curr Opin Investig Drugs* 2005, **6**(11):1131-1135.
150. Mans BJ, Ribeiro JM, Andersen JF: **Structure, function, and evolution of biogenic amine-binding proteins in soft ticks**. *The Journal of biological chemistry* 2008, **283**(27):18721-18733.
151. Beaufays J, Adam B, Menten-Dedoyart C, Fievez L, Grosjean A, Decrem Y, Prevot PP, Santini S, Brasseur R, Brossard M *et al*: **Ir-LBP, an Ixodes ricinus Tick Salivary LTB4-Binding Lipocalin, Interferes with Host Neutrophil Function**. *PLoS ONE* 2008, **3**(12).
152. Beaufays J, Adam B, Decrem Y, Prevot PP, Santini S, Brasseur R, Brossard M, Lins L, Vanhamme L, Godfroid E: ***Ixodes ricinus* Tick Lipocalins: Identification, Cloning, Phylogenetic Analysis and Biochemical Characterization**. *PLoS ONE* 2008, **3**(12).
153. Mans BJ, Ribeiro JMC: **A novel clade of cysteinyl leukotriene scavengers in soft ticks**. *Insect biochemistry and molecular biology* 2008, **38**(9):862-870.
154. Mans BJ, Ribeiro JMC: **Function, mechanism and evolution of the moubatin-clade of soft tick lipocalins**. *Insect biochemistry and molecular biology* 2008, **38**(9):841-852.
155. Mans BJ, Ribeiro JMC, Andersen JF: **Structure, function, and evolution of biogenic amine-binding proteins in soft ticks**. *Journal of Biological Chemistry* 2008, **283**(27):18721-18733.
156. Andersen JF: **Structure and mechanism in salivary proteins from blood-feeding arthropods**. *Toxicon* 2010, **56**(7):1120-1129.
157. Diaz-Martin V, Manzano-Roman R, Siles-Lucas M, Oleaga A, Perez-Sanchez R: **Cloning, characterization and diagnostic performance of the salivary lipocalin protein TSGP1 from Ornithodoros moubata**. *Veterinary parasitology* 2011, **178**(1-2):163-172.
158. Konnai S, Nishikado H, Yamada S, Imamura S, Ito T, Onuma M, Murata S, Ohashi K: **Molecular identification and expression analysis of lipocalins from blood feeding taiga tick, Ixodes persulcatus Schulze**. *Experimental parasitology* 2011, **127**(2):467-474.
159. Tirloni L, Reck J, Terra RMS, Martins JR, Mulenga A, Sherman NE, Fox JW, Yates JR, Termignoni C, Pinto AFM *et al*: **Proteomic Analysis of Cattle Tick Rhipicephalus (Boophilus) microplus Saliva: A Comparison between Partially and Fully Engorged Females**. *PLoS ONE* 2014, **9**(4).
160. Wang YA, Li Z, Zhou YZ, Cao J, Zhang HS, Gong HY, Zhou JL: **Specific histamine binding activity of a new lipocalin from Hyalomma asiaticum (Ixodidae) and therapeutic effects on allergic asthma in mice**. *Parasite Vector* 2016, **9**.
161. Manzano-Roman R, Diaz-Martin V, Oleaga A, Obolo-Mvoulouga P, Perez-Sanchez R: **TSGP4 from Ornithodoros moubata: molecular cloning, phylogenetic analysis and vaccine efficacy of a new member of the lipocalin clade of cysteinyl leukotriene scavengers**. *Veterinary parasitology* 2016, **227**:130-137.
162. Neelakanta G, Sultana H, Sonenshine DE, Andersen JF: **Identification and characterization of a histamine-binding lipocalin-like molecule from the relapsing fever tick Ornithodoros turicata**. *Insect molecular biology* 2018, **27**(2):177-187.
163. Preston SG, Majtan J, Kouremenou C, Rysnik O, Burger LF, Cruz AC, Guzman MC, Nunn MA, Paesen GC, Nuttall PA *et al*: **Novel Immunomodulators from Hard Ticks Selectively Reprogramme Human Dendritic Cell Responses**. *Plos Pathogens* 2013, **9**(6).
164. Roversi P, Johnson S, Preston SG, Nunn MA, Paesen GC, Austyn JM, Nuttall PA, Lea SM: **Structural basis of cholesterol binding by a novel clade of dendritic cell modulators from ticks**. *Scientific reports* 2017, **7**.
165. Narasimhan S, Koski RA, Beaulieu B, Anderson JF, Ramamoorthi N, Kantor F, Cappello M, Fikrig E: **A novel family of anticoagulants from the saliva of Ixodes scapularis**. *Insect molecular biology* 2002, **11**(6):641-650.
166. Narasimhan S, Montgomery RR, DePonte K, Tschudi C, Marcantonio N, Anderson JF, Sauer JR, Cappello M, Kantor FS, Fikrig E: **Disruption of Ixodes scapularis anticoagulation by using RNA interference**. *Proceedings of the National Academy of Sciences of the United States of America* 2004, **101**(5):1141-1146.
167. Assumpcao TC, Mizurini DM, Ma DY, Monteiro RQ, Ahlstedt S, Reyes M, Kotsyfakis M, Mather TN, Andersen JF, Lukszo J *et al*: **Ixonnexin from Tick Saliva Promotes Fibrinolysis by Interacting with Plasminogen and Tissue-Type Plasminogen Activator, and Prevents Arterial Thrombosis**. *Scientific reports* 2018, **8**.
168. Wagemakers A, Coumou J, Schuijt TJ, Oei A, Nijhof AM, van't Veer C, van der Poll T, Bins AD, Hovius JWR: **An Ixodes ricinus Tick Salivary Lectin Pathway Inhibitor Protects Borrelia burgdorferi sensu lato from Human Complement**. *Vector-Borne Zoonot* 2016, **16**(4):223-228.
169. Bergman DK, Ramachandra RN, Wikel SK: **Characterization of an immunosuppressant protein from Dermacentor andersoni (Acari : Ixodidae) salivary glands**. *Journal of medical entomology* 1998, **35**(4):505-509.
170. Bergman DK, Palmer MJ, Caimano MJ, Radolf JD, Wikel SK: **Isolation and molecular cloning of a secreted immunosuppressant protein from Dermacentor andersoni salivary gland**. *Journal of Parasitology* 2000, **86**(3):516-525.
171. Alarcon-Chaidez FJ, Muller-Doblies UU, Wikel S: **Characterization of a recombinant immunomodulatory protein from the salivary glands of Dermacentor andersoni**. *Parasite immunology* 2003, **25**(2):69-77.
172. Frauenschuh A, Power CA, Deruaz M, Ferreira BR, Silva JS, Teixeira MM, Dias JM, Martin T, Wells TNC, Proudfoot AEI: **Molecular cloning and characterization of a highly selective chemokine-binding protein from the tick Rhipicephalus sanguineus**. *Journal of Biological Chemistry* 2007, **282**(37):27250-27258.
173. Deruaz M, Frauenschuh A, Alessandri AL, Dias JM, Coelho FM, Russo RC, Ferreira BR, Graham GJ, Shaw JP, Wells TN *et al*: **Ticks produce highly selective chemokine binding proteins with antiinflammatory activity**. *The Journal of experimental medicine* 2008, **205**(9):2019-2031.
174. Dias JM, Losberger C, Deruaz M, Power CA, Proudfoot AEI, Shaw JP: **Structural Basis of Chemokine Sequestration by a Tick Chemokine Binding Protein: The Crystal Structure of the Complex between Evasin-1 and CCL3**. *PLoS ONE* 2009, **4**(12).
175. Vancova I, Hajnicka V, Slovak M, Kocakova P, Paesen GC, Nuttall PA: **Evasin-3-like anti-chemokine activity in salivary gland extracts of ixodid ticks during blood-feeding: a new target for tick control**. *Parasite immunology* 2010, **32**(6):460-463.
176. Bonvin P, Power CA, Proudfoot AEI: **Evasins: Therapeutic Potential of a New Family of Chemokine-Binding Proteins from Ticks**. *Frontiers in immunology* 2016, **7**.
177. Hayward J, Sanchez J, Perry A, Huang C, Valle MR, Canals M, Payne RJ, Stone MJ: **Ticks from diverse genera encode chemokine-inhibitory evasin proteins**. *Journal of Biological Chemistry* 2017, **292**(38):15670-15680.
178. Singh K, Davies G, Alenazi Y, Eaton JRO, Kawamura A, Bhattacharya S: **Yeast surface display identifies a family of evasins from ticks with novel polyvalent CC chemokine-binding activities**. *Scientific reports* 2017, **7**.
179. Alenazi Y, Singh K, Davies G, Eaton JRO, Elders P, Kawamura A, Bhattacharya S: **Genetically engineered two-warhead evasins provide a method to achieve precision targeting of disease-relevant chemokine subsets**. *Scientific reports* 2018, **8**.
180. Tyson K, Elkins C, Patterson H, Fikrig E, de Silva A: **Biochemical and functional characterization of Salp20, an Ixodes scapularis tick salivary protein that inhibits the complement pathway**. *Insect molecular biology* 2007, **16**(4):469-479.
181. Daix V, Schroeder H, Praet N, Georgin JP, Chiappino I, Gillet L, de Fays K, Decrem Y, Leboulle G, Godfroid E *et al*: **Ixodes ticks belonging to the Ixodes ricinus complex encode a family of anticomplement proteins**. *Insect molecular biology* 2007, **16**(2):155-166.
182. Soares CAG, Lima CMR, Dolan MC, Piesman J, Beard CB, Zeidner NS: **Capillary feeding of specific dsRNA induces silencing of the isac gene in nymphal Ixodes scapularis ticks**. *Insect molecular biology* 2005, **14**(4):443-452.
183. Valenzuela JG, Charlab R, Mather TN, Ribeiro JM: **Purification, cloning, and expression of a novel salivary anticomplement protein from the tick, *Ixodes scapularis***. *J Biol Chem* 2000, **275**(25):18717-18723.
184. Karczewski J, Endris R, Connolly TM: **Disagregin Is a Fibrinogen Receptor Antagonist Lacking the Arg-Gly-Asp Sequence from the Tick, Ornithodoros-Moubata**. *Journal of Biological Chemistry* 1994, **269**(9):6702-6708.
185. Wang XN, Coons LB, Taylor DB, Stevens ES, Gartner TK: **Variabilin, a novel RGD-containing antagonist of glycoprotein IIb-IIIa and platelet aggregation inhibitor from the hard tick Dermacentor variabilis**. *Journal of Biological Chemistry* 1996, **271**(30):17785-17790.
186. Mans BJ, Andersen JF, Schwan TG, Ribeiro JMC: **Characterization of anti-hemostatic factors in the argasid, Argas monolakensis: Implications for the evolution of blood-feeding in the soft tick family**. *Insect biochemistry and molecular biology* 2008, **38**(1):22-41.
