# Supplementary material for: TickSialoFam (TSFam): A Database That Helps to Classify Tick Salivary Proteins, a Review on Tick Salivary Protein Function and Evolution, With Considerations on the Tick Sialome Switching Phenomenon
Source: Front Cell Infect Microbiol. 2020 Jul 24;10:374. doi: 10.3389/fcimb.2020.00374 (PMC7396615; doi:10.3389/fcimb.2020.00374)
Supplement: Supplementary file 2 [file Data_Sheet_2.PDF]

Supplemental Table 2: Salivary proteins from ticks with a SignalP indicative of secretion but possibly having a housekeeping function.

Group	No. sequences	Argasidae	Prostriate	Amblyomminae	Haemaphysalinae	Hyalomminae	Rhipicephalinae
Immunity related							
Alpha2Macroblobulin	65	2	6	4	10	2	41
alpha-2-macroglobulin receptor	8			4	1	1	2
autophagy-related 27	6		1	3			2
Enzymes							
Glycosyltransferase	182	20	43	29	15	12	63
Glycosidase	161	16	24	14	15	7	85
Cytochrome_P450	140	19	42	6	23	3	47
Dehydrogenase	120	7	60	19	12	3	19
Disulphide isomerase	107	11	15	38	6	3	34
Coesterase	70	7	12	4	13	4	30
GILT, Gamma interferon inducible lysosomal thiol reductase	53	2	13	29	4		5
DNAJ	50	9	8	13	3	3	14
Epoxide hydrolase	34	2	3	6	2	2	19
Asparaginyl peptidase	33	4	2	4	10		13
Glycolate oxidase	30		1	24	1		4
Prolyl 4-hydroxylase alpha subunit	29	1	22	1	1		4
Arylsulfatase b-like	28	5	6	2	6		9
Triglyceride lipase-cholesterol esterase	26	1	2	5	4	1	13
Serine/threonine phosphatase	24	4	1	10	2		7
Acid phosphatase	23	1		6	4		12
Glucose dehydrogenase	22	2		12	1	2	5
Alkaline phosphatase	21		3	5	3	1	9
Thioredoxin peroxidase	19	1	2	8	2		6
Biotinidase and vanin	18	3	2	5	1	1	6
Long-chain-fatty-acid--CoA ligase 3	15			3	1		11
Furin	14	3	2		1	2	6
Carbonic anhydrase	14	2	2	3	1	1	5
Glutaredoxin	14		3	7	1		3
Nucleoside diphosphate kinase	13	2	3	4	1		3
Asparaginase	11	1	3	3	1		3
Molybdopterin cofactor sulfurase	11	2	3	3			3
Protease-associated (PA) domain	11	1		3	1	1	5
succinate-semiAldehyde dehydrogenase	10	2		3	1	1	3
FKBP-type peptidyl-prolyl cis-trans isomerase	10		3	3		1	3
GATase1_Hsp31_like	10		1	5		1	3
Glutaminyl cyclase	10	1	1	1	1	1	5
guanylate kinase	10	1	1	3			5
Tyrosine sulfotransferase	10	1		2	1	1	5
E3 ubiquitin ligase	9		2	2	1		4
Peptidylglycine alpha-amidating monooxygenase	9	1	2		2	1	3
Undecaprenyl diphosphate synthase	9		2	1	1		5
DTDP-glucose 4-6-dehydratase/udp-glucuronic acid decarboxylase	8	1	2	2		1	2
Gamma-glutamyltranspeptidase	8		1		2		5
Glycerol-3-phosphate acyltransferase 1	7	1	1	1		1	3
Acid sphingomyelinase	6	1	1		3		1
Other							
Reeler	43	2	16	8	6	2	9
Protein quiver	42	4	5	9	6	4	14
vitellogenin	42		3	7	2	1	29
Cop-coated vesicle membrane p24	28	5	5	4	3	1	10
Cyclophylin	22	4	4	9	1		4
HSP-70	17	3	2	8	2		2
Semaphorin	16		2	1	3	1	9
signal sequence receptor beta	15	3	2	6	1		3
Methyltransferase-like protein	14		4	2	5	2	1
Protein Skeletor	14		3	2	1		8
Armet	13	2	2	6	1	1	1
DDRGK	11	1	1	6			3
Cation-dependent mannose-6-phosphate receptor	10		1	5	1		3
Endoplasmic reticulum chaperone sil1 log	10	1	1	2	1	1	4
Lamp, Lysosome-associated membrane glycoprotein (Lamp)	10		1	7			2
Renin receptor-like protein	10	1	2	2	1		4
DUF2181	9		5		1		3
Endoplasmic reticulum lectin 1	9	1	1	2	1		4
Syndecan	9		1	5	1		2
ADP,ATP carrier protein	8		2	3			3
Endoplasmic reticulum oxidoreductin-1-like, ero1-like	8	1	1	3			3
Conserved secreted protein	7						7
cytochrome c oxidase subunit vic	7		4	2		1	
GILT, Gamma interferon inducible lysosomal thiol reductase	7		7				
Protocadherin-like	7		1	1			5
RGMRepulsin	7		1	2	1		3
39S ribosomal protein L44, putative	6			2			4
ER membrane protein complex subunit 7	6		1	3	1		1
G protein alpha subunit	6		1			2	3
Vitronectin receptor	6	1	2			2	1
transcription initiation factor TFIID subunit 8	5		3	1			1
ZincFinger	5		1				4
Apelin	4		4				
Cation-independent mannose-6-phosphate receptor	4		2	1			1
Replication factor C subunit 3	4			2			2
CD36	2	1		1			
Total	1,931	167	392	412	197	75	688
