# Supplementary material for: TickSialoFam (TSFam): A Database That Helps to Classify Tick Salivary Proteins, a Review on Tick Salivary Protein Function and Evolution, With Considerations on the Tick Sialome Switching Phenomenon
Source: Front Cell Infect Microbiol. 2020 Jul 24;10:374. doi: 10.3389/fcimb.2020.00374 (PMC7396615; doi:10.3389/fcimb.2020.00374)

Supplemental figure 1: Molecular Phylogenetic analysis of the tick salivary 5'-nucleotidase/Apyrase protein-coding genes. The evolutionary history was inferred by using the Maximum Likelihood method based on the Tamura-Nei model [1]. The tree with the highest log likelihood (-14996.10) is shown. The percentage of trees in which the associated taxa clustered together is shown next to the branches. Initial tree(s) for the heuristic search were obtained automatically by applying Neighbor-Join and BioNJ algorithms to a matrix of pairwise distances estimated using the Maximum Composite Likelihood (MCL) approach, and then selecting the topology with superior log likelihood value. The tree is drawn to scale, with branch lengths measured in the number of substitutions per site. The analysis involved 50 nucleotide sequences. Codon positions included were 1st. All positions with less than 50% site coverage were eliminated. That is, fewer than 50% alignment gaps, missing data, and ambiguous bases were allowed at any position. There were a total of 584 positions in the final dataset. Evolutionary analyses were conducted in MEGA7 [2]. The tree was rooted on the GPI-containing branch. Red branches represent those containing only *Ixodes* sequences, while the green one is Argasidae exclusive.

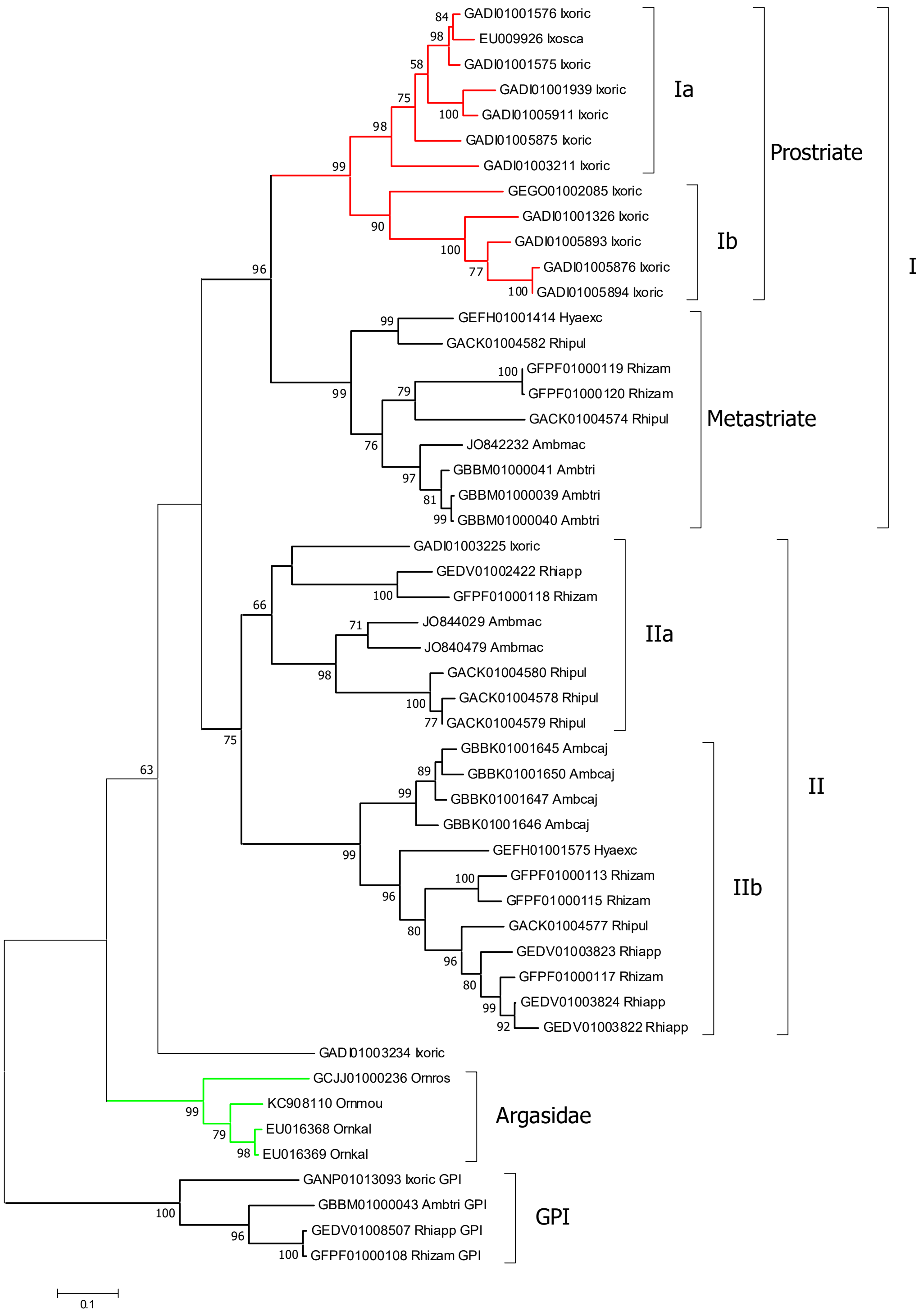

Supplemental figure 2: Molecular Phylogenetic analysis of a subset of *Ixodes ricinus* sequences coding for lipocalins containing thePfam His_bind motif. The evolutionary history was inferred by using the Maximum Likelihood method based on the Tamura-Nei model [1]. The tree with the highest log likelihood (-9433.11) is shown. The percentage of trees in which the associated taxa clustered together is shown next to the branches. Initial tree(s) for the heuristic search were obtained automatically by applying Neighbor-Join and BioNJ algorithms to a matrix of pairwise distances estimated using the Maximum Composite Likelihood (MCL) approach, and then selecting the topology with superior log likelihood value. The tree is drawn to scale, with branch lengths measured in the number of substitutions per site. The analysis involved 32 nucleotide sequences. All positions with less than 50% site coverage were eliminated. That is, fewer than 50% alignment gaps, missing data, and ambiguous bases were allowed at any position. There were a total of 561 positions in the final dataset. Evolutionary analyses were conducted in MEGA7 [2].

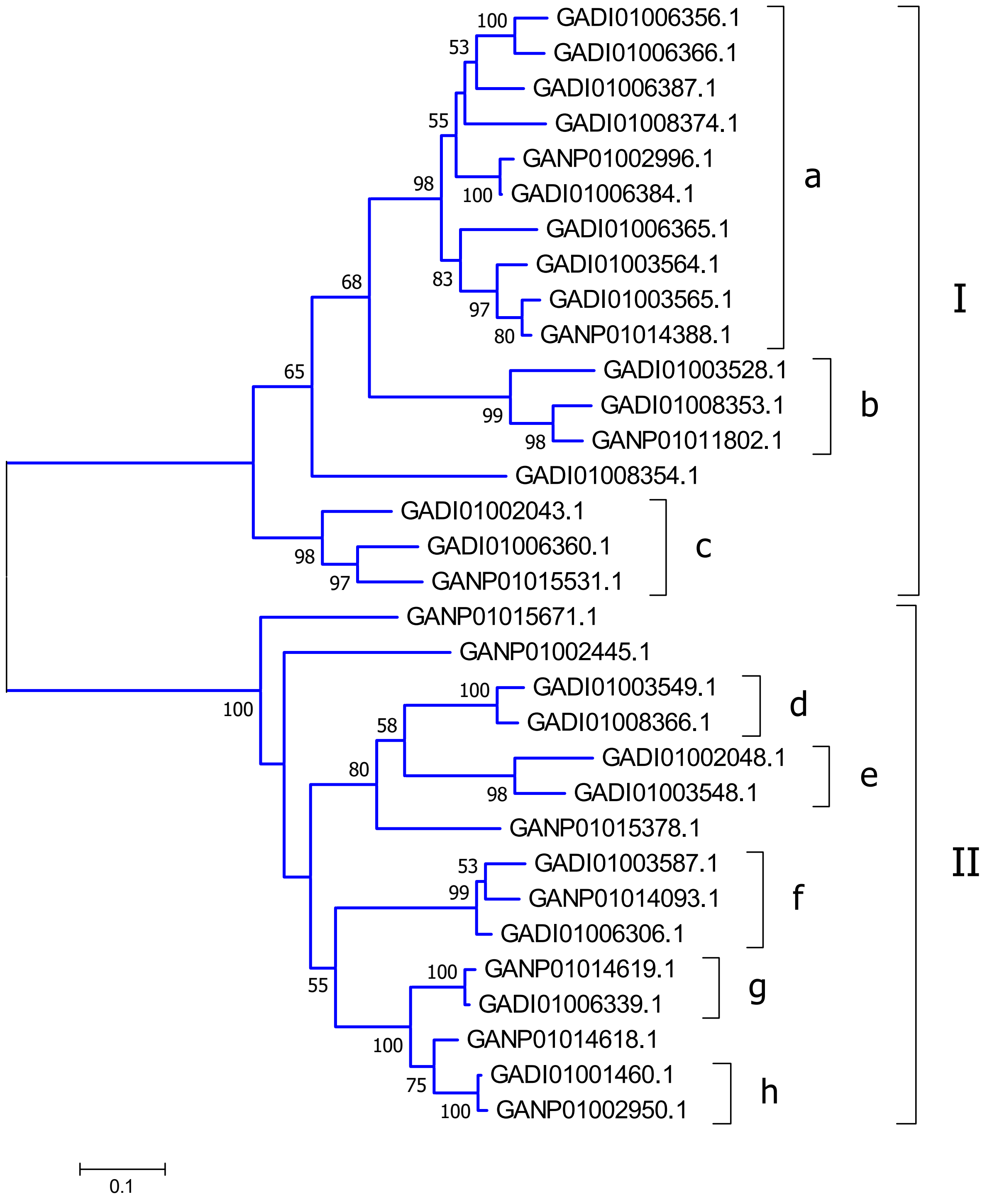

Supplemental figure 3: Phylogenetic reconstruction of the tick salivary metalloprotease coding genes, containing the CDD domain cd04272 named ZnMc_salivary_gland_MPs. The evolutionary history was inferred using the Neighbor-Joining method [1]. The bootstrap consensus tree inferred from 1000 replicates [2] is taken to represent the evolutionary history of the taxa analyzed [2]. Branches corresponding to partitions reproduced in less than 50% bootstrap replicates are collapsed. The percentage of replicate trees in which the associated taxa clustered together in the bootstrap test (1000 replicates) are shown next to the branches [2]. The evolutionary distances were computed using the Maximum Composite Likelihood method [3] and are in the units of the number of base substitutions per site. The analysis involved 87 nucleotide sequences. Codon positions included were 1st+2nd+3rd. All positions with less than 50% site coverage were eliminated. That is, fewer than 50% alignment gaps, missing data, and ambiguous bases were allowed at any position. There were a total of 1530 positions in the final dataset. Evolutionary analyses were conducted in MEGA7 [4].

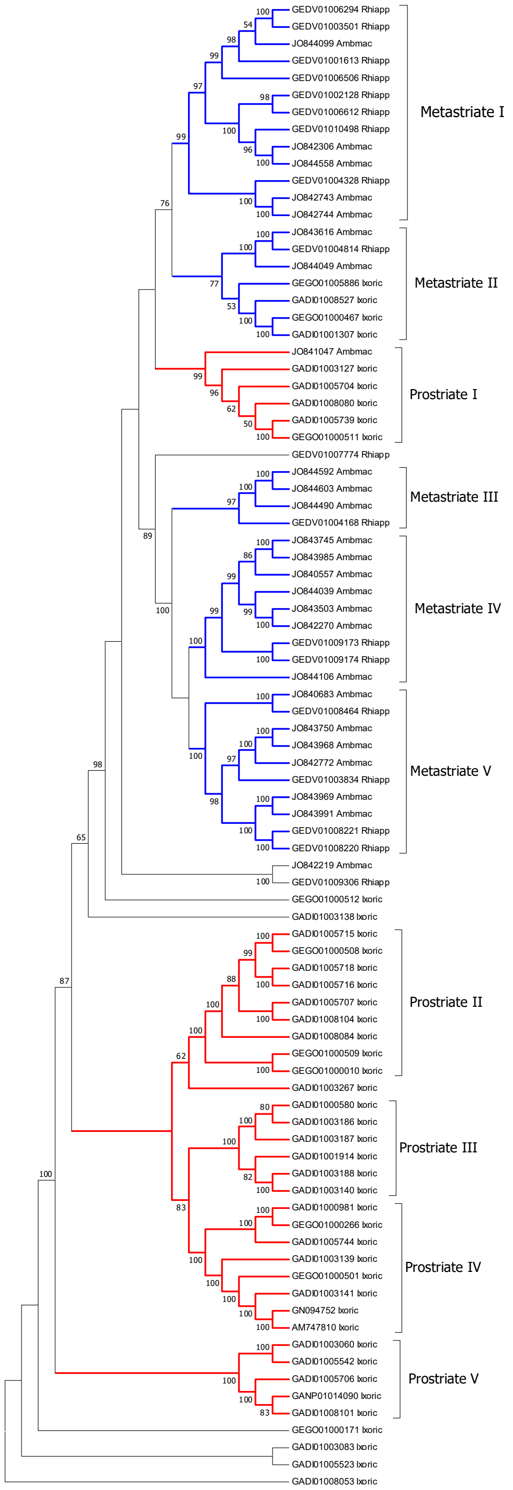

Supplemental figure 4: Phylogenetic reconstruction of the tick salivary DAP-36 coding genes. The evolutionary history was inferred using the Neighbor-Joining method [1]. The optimal tree with the sum of branch length = 36.20446236 is shown. The percentage of replicate trees in which the associated taxa clustered together in the bootstrap test (1000 replicates) are shown next to the branches [2]. The tree is drawn to scale, with branch lengths in the same units as those of the evolutionary distances used to infer the phylogenetic tree. The evolutionary distances were computed using the Maximum Composite Likelihood method [3] and are in the units of the number of base substitutions per site. The analysis involved 120 nucleotide sequences. Codon positions included were 1st. All positions with less than 50% site coverage were eliminated. That is, fewer than 50% alignment gaps, missing data, and ambiguous bases were allowed at any position. There were a total of 248 positions in the final dataset. Evolutionary analyses were conducted in MEGA7 [4].

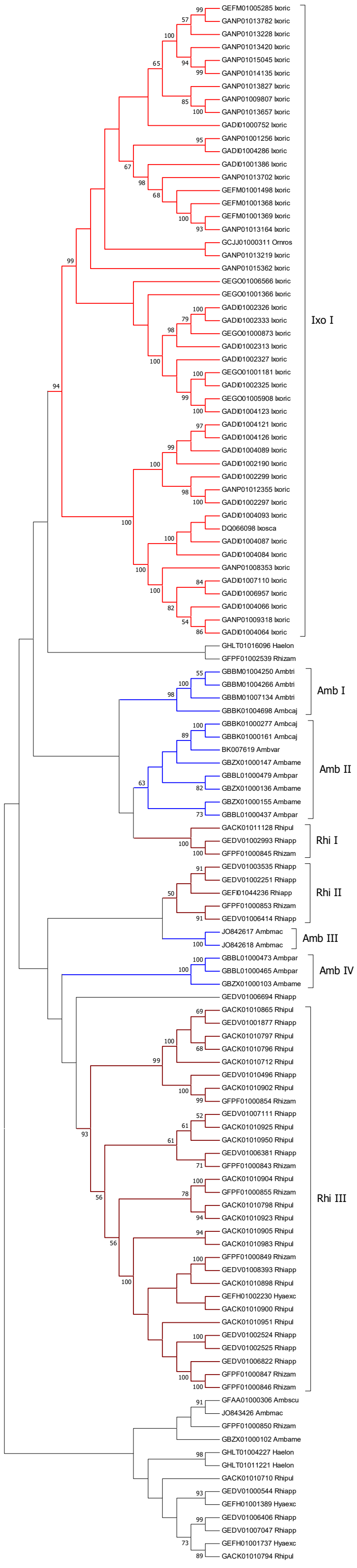

Supplemental figure 5: Phylogenetic reconstruction of the tick salivary cystatins. The evolutionary history was inferred using the Neighbor-Joining method [1]. The bootstrap consensus tree inferred from 10000 replicates [4] is taken to represent the evolutionary history of the taxa analyzed [4]. Branches corresponding to partitions reproduced in less than 50% bootstrap replicates are collapsed. The percentage of replicate trees in which the associated taxa clustered together in the bootstrap test (10000 replicates) are shown next to the branches [4]. The evolutionary distances were computed using the Maximum Composite Likelihood method [2] and are in the units of the number of base substitutions per site. The analysis involved 162 nucleotide sequences. All ambiguous positions were removed for each sequence pair. There were a total of 717 positions in the final dataset. Evolutionary analyses were conducted in MEGA7 [3].

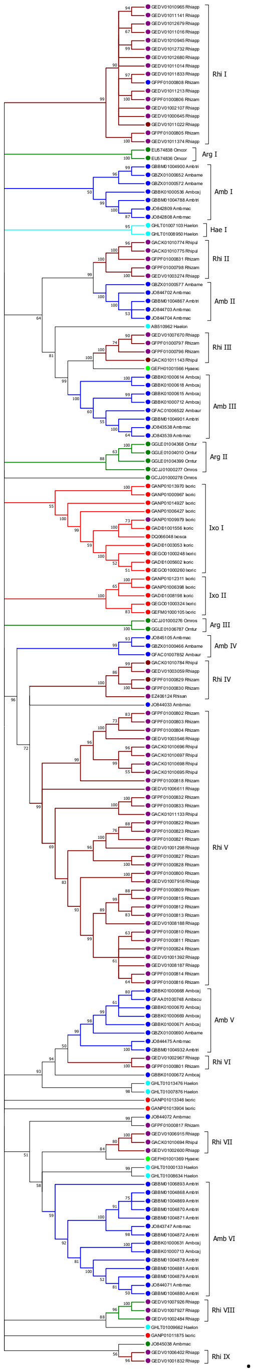

Supplemental figure 6: Phylogenetic reconstruction of the tick subolesins. The evolutionary history was inferred by using the Maximum Likelihood method based on the Tamura-Nei model [1]. The bootstrap consensus tree inferred from 1000 replicates [3] is taken to represent the evolutionary history of the taxa analyzed [3]. Branches corresponding to partitions reproduced in less than 50% bootstrap replicates are collapsed. The percentage of replicate trees in which the associated taxa clustered together in the bootstrap test (1000 replicates) are shown next to the branches [3]. Initial tree(s) for the heuristic search were obtained automatically by applying Neighbor-Join and BioNJ algorithms to a matrix of pairwise distances estimated using the Maximum Composite Likelihood (MCL) approach, and then selecting the topology with superior log likelihood value. The analysis involved 16 nucleotide sequences. Codon positions included were 1st+2nd+3rd+Noncoding. All positions with less than 50% site coverage were eliminated. That is, fewer than 50% alignment gaps, missing data, and ambiguous bases were allowed at any position. There were a total of 531 positions in the final dataset. Evolutionary analyses were conducted in MEGA7 [2].

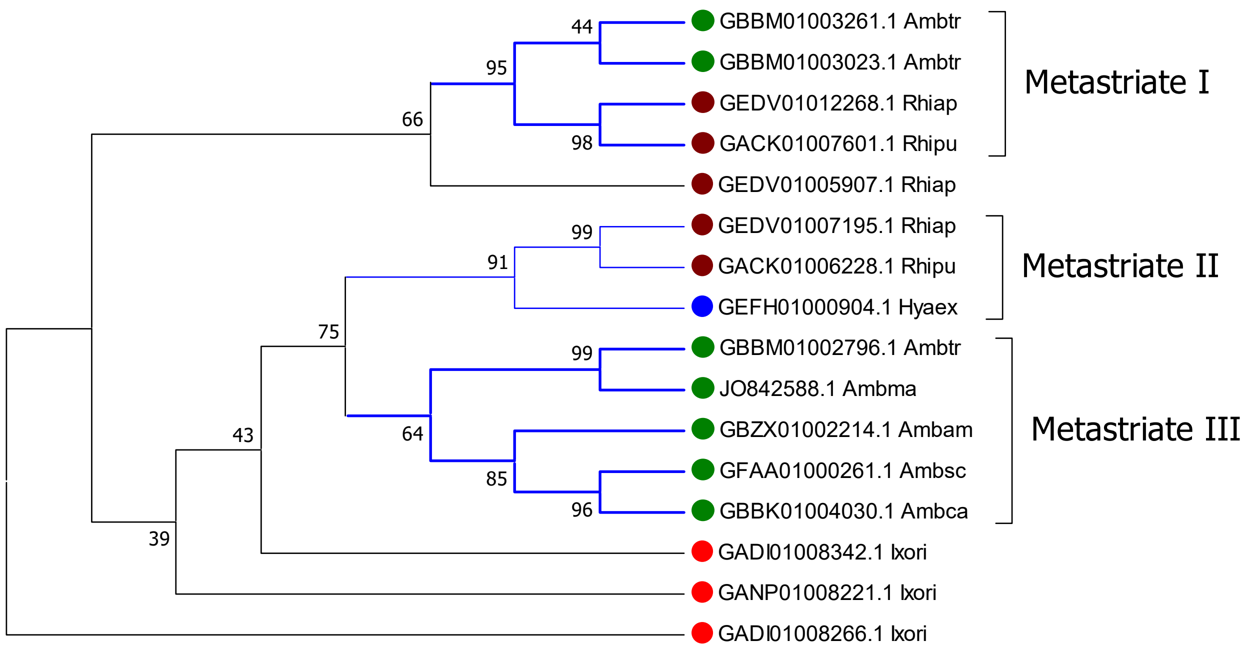

Supplement: Supplementary file 3 [file Data_Sheet_3.PDF]
