# Supplementary material for: TickSialoFam (TSFam): A Database That Helps to Classify Tick Salivary Proteins, a Review on Tick Salivary Protein Function and Evolution, With Considerations on the Tick Sialome Switching Phenomenon
Source: Front Cell Infect Microbiol. 2020 Jul 24;10:374. doi: 10.3389/fcimb.2020.00374 (PMC7396615; doi:10.3389/fcimb.2020.00374)
Supplement: Supplementary file 4 [file Table_1.DOCX]

**Supplemental files**

Link to supplemental spreadsheets 1-3

<https://proj-bip-prod-publicread.s3.amazonaws.com/transcriptome/TickSialoFam/Supplemental_spreadaheets.zip>

Link to TickSialoFam database

<https://proj-bip-prod-publicread.s3.amazonaws.com/transcriptome/TickSialoFam/TSF-RPS.zip>
